# Effects of Community-Wide Vaccination with PCV-7 on Pneumococcal Nasopharyngeal Carriage in The Gambia: A Cluster-Randomized Trial

**DOI:** 10.1371/journal.pmed.1001107

**Published:** 2011-10-18

**Authors:** Anna Roca, Philip C. Hill, John Townend, Uzo Egere, Martin Antonio, Abdoulie Bojang, Abiodun Akisanya, Teresa Litchfield, David E. Nsekpong, Claire Oluwalana, Stephen R. C. Howie, Brian Greenwood, Richard A. Adegbola

**Affiliations:** 1 Medical Research Council Unit, The Gambia; 2 Barcelona Center for International Health Research (CRESIB), Institut d'Investigacions Biomediques August Pi i Sunyer, Universitat de Barcelona, Barcelona, Spain; 3 Centre for International Health, School of Medicine, University of Otago, Dunedin, New Zealand; 4 Faculty of Infectious and Tropical Diseases, London School of Hygiene & Tropical Medicine, London, United Kingdom; 5 Bill & Melinda Gates Foundation, Seattle, Washington, United States of America; Health Protection Agency, Centre for Infections, United Kingdom

## Abstract

**Background:**

Introduction of pneumococcal conjugate vaccines (PCVs) of limited valency is justified in Africa by the high burden of pneumococcal disease. Long-term beneficial effects of PCVs may be countered by serotype replacement. We aimed to determine the impact of PCV-7 vaccination on pneumococcal carriage in rural Gambia.

**Methods and Findings:**

A cluster-randomized (by village) trial of the impact of PCV-7 on pneumococcal nasopharyngeal carriage was conducted in 21 Gambian villages between December 2003 to June 2008 (5,441 inhabitants in 2006). Analysis was complemented with data obtained before vaccination. Because efficacy of PCV-9 in young Gambian children had been shown, it was considered unethical not to give PCV-7 to young children in all of the study villages. PCV-7 was given to children below 30 mo of age and to those born during the trial in all study villages. Villages were randomized (older children and adults) to receive one dose of PCV-7 (11 vaccinated villages) or meningococcal serogroup C conjugate vaccine (10 control villages). Cross-sectional surveys (CSSs) to collect nasopharyngeal swabs were conducted before vaccination (2,094 samples in the baseline CSS), and 4–6, 12, and 22 mo after vaccination (1,168, 1,210, and 446 samples in CSS-1, -2, and -3, respectively).

A time trend analysis showed a marked fall in the prevalence of vaccine-type pneumococcal carriage in all age groups following vaccination (from 23.7% and 26.8% in the baseline CSS to 7.1% and 8.5% in CSS-1, in vaccinated and control villages, respectively). The prevalence of vaccine-type pneumococcal carriage was lower in vaccinated than in control villages among older children (5 y to <15 y of age) and adults (≥15 y of age) at CSS-2 (odds ratio [OR] = 0.15 [95% CI 0.04–0.57] and OR = 0.32 [95% CI 0.10–0.98], respectively) and at CSS-3 (OR = 0.37 [95% CI 0.15–0.90] for older children, and 0% versus 7.6% for adults in vaccinated and control villages, respectively). Differences in the prevalence of non-vaccine-type pneumococcal carriage between vaccinated and control villages were small.

**Conclusions:**

Vaccination of Gambian children reduced vaccine-type pneumococcal carriage across all age groups, indicating a “herd effect” in non-vaccinated older children and adults. No significant serotype replacement was detected.

*Please see later in the article for the Editors' Summary*

## Abstract 2

In a cluster-randomized trial conducted in Gambian villages, Anna Roca and colleagues find that vaccination of children with pneumococcal conjugate vaccines reduced vaccine-type pneumococcal carriage even among nonvaccinated older children and adults.

## Editors' Summary

**Background:**

The prevention of pneumococcal disease, especially in children in developing countries, is a major international public health priority. Despite all the international attention on the UN's Millennium Development Goal 4—to reduce deaths in children under five years by two-thirds between 1990 and 2015—pneumonia, sepsis, and meningitis together compose more than 25% of the 10 million deaths occurring in children less than five years of age. *Streptococcus pneumoniae* is a leading bacterial cause of these diseases, and the World Health Organization estimates that approximately 800,000 children die each year of invasive pneumococcal disease.

Pneumococcal conjugate vaccines are currently available and protect against the serotypes that most commonly cause invasive pneumococcal disease in young children in North America and Europe. Such vaccines have been highly successful in reducing the incidence of invasive pneumococcal disease in both vaccinated children and in the non-vaccinated older population by reducing nasopharyngeal carriage (presence of pneumococcal bacteria in the back of the nose) in vaccinated infants, resulting in decreased transmission to contacts—the so-called herd effect. However, few countries with the highest burden of invasive pneumococcal disease, especially those in sub-Saharan Africa, have introduced the vaccine into their national immunization programs.

**Why Was This Study Done?:**

The features of pneumococcal nasopharyngeal carriage and invasive pneumococcal disease in sub-Saharan Africa are different than in other regions. Therefore, careful evaluation of the immune effects of vaccination requires long-term, longitudinal studies. As an alternative to such long-term observational studies, and to anticipate the potential long-term effects of the introduction of pneumococcal conjugate vaccination in sub-Saharan Africa, the researchers conducted a cluster-randomized (by village) trial in The Gambia in which the whole populations of some villages were immunized with the vaccine PCV-7, and other villages received a control.

**What Did the Researchers Do and Find?:**

With full consent from communities, the researchers randomized 21 similar villages in a rural region of western Gambia to receive pneumococcal conjugate vaccine or a control—meningococcal serogroup C conjugated vaccine, which is unlikely to affect pneumococcal carriage rates. For ethical reasons, the researchers only randomized residents aged over 30 months—all young infants received PCV-7, as a similar vaccine had already been shown to be effective in young infants. Before immunization began, the researchers took nasopharyngeal swabs from a random selection of village residents to determine the baseline pneumococcal carriage rates of both the serotypes of pneumococci covered by the vaccine (vaccine types, VTs) and the serotypes of pneumococci not covered in the vaccine (non-vaccine types, NVTs). The researchers then took nasopharyngeal swabs from a random sample of 1,200 of village residents in both groups of villages in cross-sectional surveys at 4–6, 12, and 22 months after vaccination. Villagers and laboratory staff were unaware of which vaccine was which (that is, they were blinded).

Before immunization, the overall prevalence of pneumococcal carriage in both groups was high, at 71.1%, and decreased with age. After vaccination, the overall prevalence of pneumococcal carriage in all three surveys was similar between vaccinated and control villages, showing a marked fall. However, the prevalence of carriage of VT pneumococci was significantly lower in vaccinated than in control villages in all surveys for all age groups. The prevalence of carriage of NVT pneumococci was similar in vaccinated and in control villages, except for a slightly higher prevalence of NVT pneumococci among vaccinated communities in adults at 4–6 months after vaccination. The researchers also found that the overall prevalence of pneumococcal carriage fell markedly after vaccination and reached minimum levels at 12 months in both study arms and in all age groups.

**What Do These Findings Mean?:**

These findings show that vaccination of young Gambian children reduced carriage of VT pneumococci in vaccinated children but also in vaccinated and non-vaccinated older children and adults, revealing a potential herd effect from vaccination of young children. Furthermore, the immunological pressure induced by vaccinating whole communities did not lead to a community-wide increase in carriage of NVT pneumococci during a two-year period after vaccination. The researchers plan to conduct more long-term follow-up studies to determine nasopharyngeal carriage in these communities.

**Additional Information:**

Please access these websites via the online version of this summary at http://dx.doi.org/10.1371/journal.pmed.1001107.

- The World Health Organization has information about pneumococcus
- The US Centers for Disease Control and Prevention provides information about pneumococcal conjugate vaccination

## Introduction

The prevention of pneumococcal disease is a major international public health priority, especially in children in developing countries [1]. On the basis of a trial conducted in California in the United States [2], the first licensed pneumococcal conjugate vaccine (PCV), PCV-7, was introduced in the US in 2000. This vaccine has been highly successful in reducing the incidence of invasive pneumococcal disease (IPD) in both vaccinated children and in the non-vaccinated older population [3],[4]. This herd effect has been attributed to reduced nasopharyngeal carriage of pneumococci of vaccine type (VT) in vaccinated infants, resulting in decreased transmission to contacts [3]–[6]. In the US, small increases in the incidence of both IPD and carriage caused by pneumococci of non-vaccine types (NVTs) have been detected, a phenomenon termed serotype replacement [4],[7]–[12]. In European countries, the prevalence of serotypes included in PCV-7 is generally lower than in the US, and the impact of PCV-7 has been more heterogeneous, with less overall public health benefit as a result of more marked serotype replacement [10]–[14].

Introduction of PCVs into the routine immunization programs of sub-Saharan African countries is justified by the high pneumococcal disease burden [1],[15]–[19] and the evidence of efficacy of PCVs provided by clinical trials [20],[21]. However, the ecology and epidemiology of pneumococcal carriage and IPD in this region is different from that seen in the US or Europe. First, rates of pneumococcal carriage in sub-Saharan Africa are among the highest described [22]–[25] and result in high rates of transmission [26]. Second, there is a wide variety of serotypes associated with both carriage and invasive disease, and prevalence of individual serotypes differs from those seen in developed countries [15],[22]–[28]. Third, vaccination in African countries is usually a three-dose regimen given during infancy, without a booster, as opposed to the booster-containing schedules widely used in industrialized countries.

The factors that drive serotype replacement, which vary in degree from community to community [29], are not fully understood. It is likely that vaccination facilitates serotype replacement by inducing a humoral and/or cellular immune response that makes it more difficult for VT pneumococci to colonize the nasopharynx, opening the way for colonization by NVT pneumococci and, perhaps, by other bacteria. In countries where PCV has been introduced into routine immunization programs, immune pressure against VT pneumococci will increase gradually as more vaccinated individuals enter the population. Determining the consequence of this will require long-term longitudinal studies. As an alternative, we have undertaken a village-randomized trial in which the pneumococcal population of some villages has been exposed to maximum immune pressure by immunizing the whole population with PCV-7.

## Methods

### Study Population

Villages in the Sibanor area, Western Region, The Gambia, were selected for the study as they are representative of rural areas in The Gambia and are distant from the area where a PCV-9 vaccine trial had been undertaken a few years earlier [20]. Twenty-one of the 55 villages in the Sibanor area were selected based on the criteria that they had a population ranging from 80 to 660 inhabitants and were separated from each other by a distance of at least 3 km. The overall population of the selected villages was 5,441 in June 2006. During the last trimester of 2006, 1,532 refugees from southern Senegal lived temporarily in these villages. The prevalence of HIV infection among pregnant women attending Sibanor Hospital, adjacent to the study area, between 2000 and 2001 was 3% [30] and is stable. The climate of The Gambia is tropical with one short rainy season from June to October. Other characteristics of the study population are described elsewhere [23].

### Study Preparation

Before the trial started, village meetings were held to discuss the nature and objectives of the study with village elders and other members of the study villages [23]. When village consent had been obtained, the village was mapped and a full census taken and updated annually. All residents of the 21 selected villages were eligible for inclusion in the study, including those born during the study period. Study participants gave individual informed consent; parental consent was obtained for children up to 16 y of age.

The study was approved by the joint Medical Research Council (MRC)/Gambia Government Ethics Committee and by the ethics committee of the London School of Hygiene and Tropical Medicine. The conduct of the trial was guided by a Data Safety and Monitoring Board.

### Trial Design

The protocol of the trial and CONSORT checklist are available in Text S1 and Text S2, respectively.

A cluster-randomized (by village) controlled trial of PCV-7 was conducted. PCV-7 was the study vaccine, and a meningococcal serogroup C conjugate was the control vaccine. Blinding included vaccine recipients and laboratory personnel. Study nurses were aware of the nature of the vaccines given but played no other part in the conduct of the trial.

Time trends within the vaccinated and control villages were investigated using a before and after approach, measuring pneumococcal carriage in PCV-7 vaccinated and control villages before and after vaccination.

#### Pre-vaccination cross-sectional survey

Between 8 December 2003 and 19 May 2004, 2 y before vaccination started, a baseline cross-sectional survey (CSS) was undertaken in 21 villages in the Sibanor area. A nasopharyngeal swab (NPS) was collected from a random selection of the village's population and cultured for pneumococci. Details of how this baseline study was conducted and its findings have been reported previously [23]. The overall prevalence of pneumococcal carriage among children and adults was approximately 70%. A random selection of the NPSs collected in this baseline CSS has been used to provide a population with a similar age distribution to the populations investigated in the post-vaccination CSSs described below. Sampling and culturing methods for the baseline and post-vaccination CSSs did not differ.

#### Randomization

The number of residents in the study villages varied substantially. Therefore, village randomization was performed at the MRC laboratories at Fajara using several steps. First, a random integer was chosen to select 11 or 10 intervention villages versus 10 or 11 control villages. Population sizes in January 2006 for the 21 villages were stored in a Stata program file (do file) for the village allocation scheme. One thousand allocation-size-balanced schemes (difference between the two groups of less than 10%) were created, and one was randomly chosen.

#### Vaccination

Because the non-licensed PCV-9 vaccine (including serotypes 1 and 5 in addition to the PCV-7 serotypes) had already been shown to be effective in young children in a randomized controlled trial conducted in The Gambia, it was considered unethical not to give PCV-7 to young children in all of the study villages. Therefore, all children in the trial aged between 2 and 30 mo at the start of the study, the age group covered by the PCV-9 trial [20], received PCV-7, as did any child born during the period of the study. Infants aged between 2 and 11 mo received three doses of the vaccine given at monthly intervals, and children aged between 12 and 30 mo received two doses with a 1-mo gap between injections. Infants born during the study received three doses of the vaccine given monthly at the ages of approximately 2, 3, and 4 mo.

Village randomization was applied to individuals who were above the age of 30 mo when vaccination started. On the basis of their randomization group, individuals above the age of 30 mo received either one dose of PCV-7 (Prevenar, Pfizer) (11 vaccinated villages) or one dose of a meningococcal serogroup C conjugate vaccine (Meningitec, Pfizer) (10 control villages). The meningococcal vaccine was given to ensure blinding and is not expected to have had any effect on pneumococcal carriage.

PCV-7 vaccination started in 17 July 2006, and by 2 mo the first round of vaccination had been completed (first dose given to 50%–75% of eligible individuals in vaccinated and control villages). Meningococcal vaccine was unavailable for several weeks, and hence meningococcal vaccination was delayed beyond the intended date of administration. Individuals who moved into the study villages during the study period were vaccinated and managed following the standardized procedures of the trial, including 1,532 Senegalese refugees who moved into 13 of the study villages (869 in control and 633 in vaccinated villages) during the last trimester of 2006. Following a census update, these individuals were vaccinated and managed following the study protocol. No serious adverse events that could be attributed to PCV-7 were observed in children or adults.

#### Post-vaccination surveys

Approximately 4–6, 12, and 22 mo after vaccination, a NPS sample was collected from 1,200 randomly selected individuals during CSSs (post-vaccination CSS-1 [15 November 2006–13 March 2007], CSS-2 [17 July–12 September 2007], and CSS-3 [8 July–1 September 2008], respectively). It was originally planned that CSS-3 would be conducted 24 mo after vaccination. However, a mass campaign by the Gambian National Trachoma Elimination Programme involving administration of one dose of azithromycin to all individuals older than 6 mo of age except pregnant women started at the time of the last study CSS in most study villages, meaning that the results of this survey could not ultimately be used. Instead, samples from a concurrent longitudinal survey in the same villages taken 0 to 3 mo before the intended start of the final CSS (12 April–25 June 2008) were used to estimate the effects of vaccination 2 y after the start of the trial. A maximum of one sample per individual was included from this dataset, giving a total of 446 samples that we called CSS-3. All these 446 samples were collected before the azithromycin campaign started.

### Sample Handling

NPSs were collected during all CSSs using a calcium alginate swab, from the posterior wall of the nasopharynx, and immediately inoculated into vials containing skim-milk-tryptone-glucose-glycerol transport medium, which were placed in a cold box before transfer to the MRC Fajara laboratories (a distance of 90 km) within 8 h of collection, in accordance with the World Health Organization protocol for evaluation of pneumococcal carriage [31]. Inoculated vials were stored at −70°C until they were tested in batches by subculture onto gentamicin blood agar for selective isolation of *Streptococcus pneumoniae*.

### Laboratory Methods

10 µl of thawed, inoculated skim-milk-tryptone-glucose-glycerol medium was plated onto gentamicin blood agar and incubated for 18–24 h at 35°C in 5% CO_2_. Pneumococcal identification was based on colony morphology and conventional methods of characterization (optochin susceptibility and bile solubility assays) [23]. Serotyping was performed at MRC Fajara laboratories, with capsular and factor typing sera (Statens Serum Institut), using a modified latex agglutination assay [32]. Equivocal results were confirmed by the Quellung reaction. Laboratory methods were consistent for all CSSs.

### Data Management and Statistical Analysis

Age groups were defined as follows: (i) young children: individuals 2 to 5 y of age; (ii) older children: individuals 5 to 15 y of age; and (iii) adults: individuals aged 15 y and above. Pneumococcal serotypes were grouped as follows: (i) VT: serotypes included in PCV-7 (4, 6B, 9V, 14, 18C, 19F, and 23F) plus 6A, which shows cross-protective immunity with serotype 6B; and (ii) NVT: any other pneumococcal serotype including non-typable pneumococcus. Individuals can carry more than one serotype, and all serotypes were included in the analysis. The primary outcomes of the study for each of the age groups were (i) the prevalence of carriage of VT pneumococci and (ii) the prevalence of carriage of NVT pneumococci.

The sample size was calculated so as to be able to detect a 50% reduction in the prevalence of carriage of VT for each of the three study age groups among individuals resident in PCV-7 vaccinated villages compared to individuals from control villages, with 90% power at a 5% significance level (from 30% to 15%). These calculations resulted in an overall sample size of 1,200 individuals per CSS. The study power for comparing study arms in CSS-3 was compromised by the unanticipated trachoma elimination campaign. In an ad hoc analysis, we also included comparison of individual serotypes between vaccinated and control villages for each CSS covering all study age groups. Because all children under the age of 30 mo at enrollment or born during the study received PCV-7 (in both vaccinated and control villages), the effect of vaccination on carriage could not be measured among young children by comparing vaccinated versus control villages. However, the study protocol allowed the evaluation of the indirect effect of vaccinating children on the rest of the population of the control villages. Analysis of carriage used “person” as the unit of analysis, whereas analysis of serotypes used “isolate” as the unit of analysis.

Comparisons of median ages, village populations, and other quantitative measures of household characteristics in vaccinated and control villages were made using Wilcoxon rank-sum tests. Comparisons of sex, ethnic group distribution, and other categorical variables were made using Chi-square tests. For each of the CSSs, comparisons were made of carriage of VT and NVT pneumococci in control versus vaccinated villages using mixed effects logistic regression with village included as a random effect to allow for the cluster randomized design. Mixed effects logistic regression was used to compare carriage in different CSSs within each study arm, treating CSS as a categorical variable and adjusting for age as a continuous variable. For individual serotypes, similar analyses were carried out using fixed effects logistic regression and a clustered sandwich variance estimator to allow for the clustered design. The analyses were carried out using Stata 11 (StataCorp). *p*-Values less than 0.05 were taken to indicate statistical significance.

## Results

### Trial Profile

**Figure 1:**
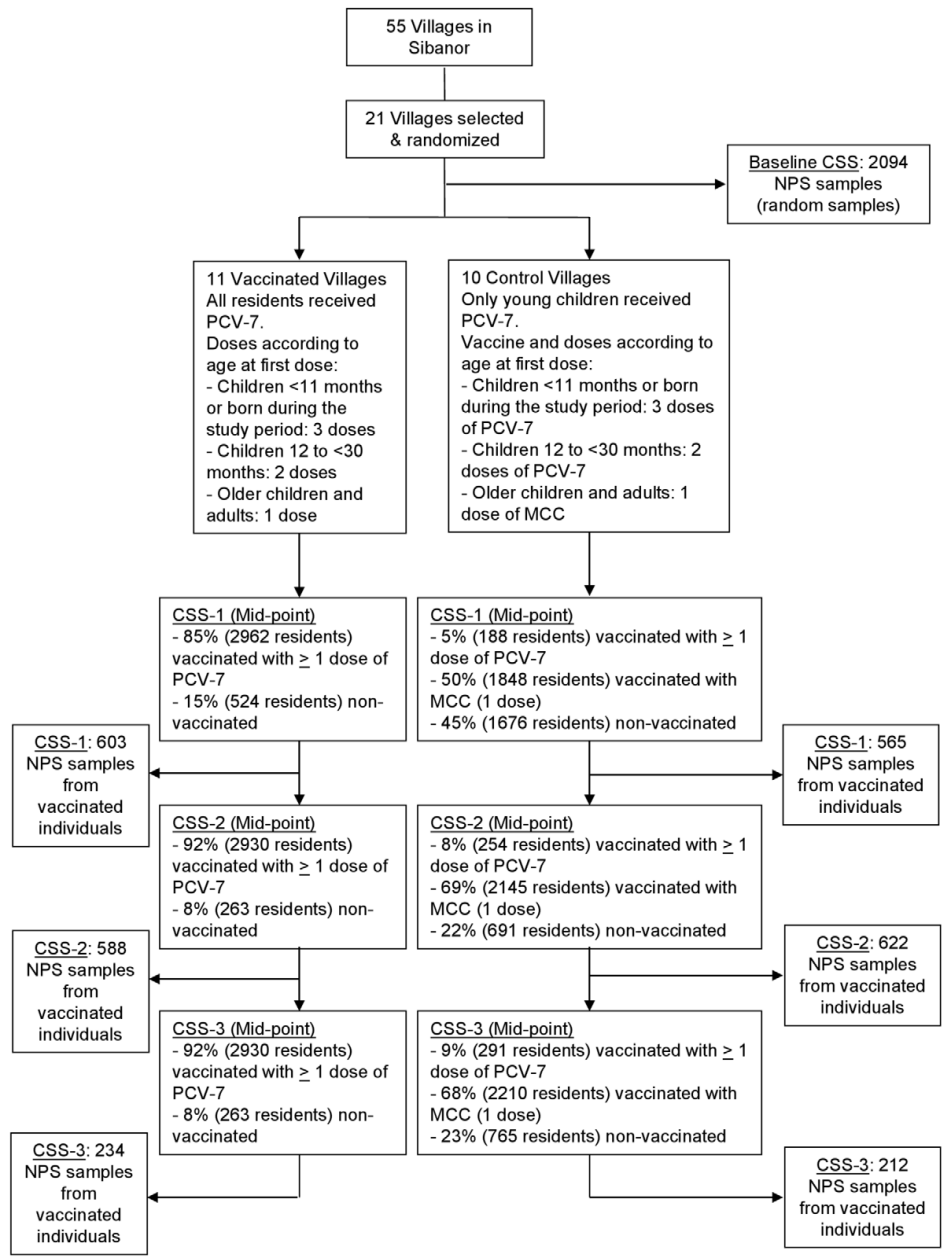
Trial profile. MCC, meningococcal polysaccharide C conjugate vaccine.

Twenty-one villages were randomized to one or the other of the two study groups (Figure 1). Characteristics of the two groups of villages (vaccinated and control) are shown in Table 1. Demographic and epidemiological characteristics of the villages were similar, except for the distribution of ethnic groups (*p*<0.001); the Mandinka ethnic group was overrepresented in the vaccinated villages. Characteristics of the individuals included in each of the study CSSs are shown in Table S1. The percentages of the population that had been vaccinated by the time of each post-vaccination CSS are shown in Figure 1 and Table S2. Vaccine coverage with PCV-7 in the vaccinated villages ranged from 85% to 92%. In the control communities, PCV-7 coverage in the post-vaccination surveys increased from 33% (CSS-1) to 62% (CSS-3) in young children, who represented 5% and 9% of the overall population in this study arm, respectively. Additional information regarding time since PCV-7 vaccination in the study villages during each of the post-vaccination CSSs is shown in Table S3. Although PCV-7 vaccination was conducted simultaneously in vaccinated and control villages, NPS collection in CSS-1 was performed slightly later in control than in vaccinated villages. In CSS-1, half of the individuals were sampled within 177 d (approximately 6 mo) after PCV-7 vaccination started in control villages and within 125 d (approximately 4 mo) after PCV-7 vaccination started in vaccinated villages. Coverage with meningococcal vaccine in older children and adults in control villages in CSS-1 was low (Figure 1 and Table S2) and improved over the CSSs, but this delay should not have any effect on the study objectives.

**Table 1:** A comparison of characteristics of the population residents at the start of the trial (17 July 2006) in the control and vaccinated villages.

Characteristic	Control Villages	Vaccinated Villages	*p*-Value
**Village populations**			
Total	2,765	2,676	
Median (range)	198 (140–528)	251 (69–634)	*p* = 0.622
Total number of babies born during the trial (17 July 2006–1 September 2008)	190	215	*p* = 0.233
**Age distribution**			
<30 m (%)	6.2	6.8	
30 m to 59 m (%)	7.4	8.3	
5 y to <15 y (%)	28.2	29.9	
15 y to <50 y (%)	45.7	41.7	
≥50 y (%)	12.4	13.3	
Median (years)	18	16	*p* = 0.372
**Sex distribution**			
Male (%)	50.1	49.8	
Female (%)	49.9	50.2	*p* = 0.796
**Median number of compounds (range)**	13 (4–27)	11 (6–37)	*p* = 0.750
**Median number of households per compound (range)**	1 (1–9)	1 (1–17)	*p* = 0.246
**Median distance from Sibanor main road (IQR)**	3 (2–4)	4 (2–6)	*p* = 0.422
**Median distance from the closest health center (IQR)**	9.5 (5–12)	6 (3–13)	*p* = 0.447
**Cooking fuel**			
Collect firewood (%)	95.3	91	*p* = 0.041
Buy firewood (%)	1.8	10.1	*p*≤0.001
Crop residues (%)	1.5	1.4	*p* = 1.000
Charcoal (%)	1.8	1.2	*p* = 0.519
Gas (%)	1.5	1.7	*p* = 1.000

IQR, interquartile range.

Overall, 2,094 NPS samples were included in the pre-vaccination CSS and 1,168, 1,210, and 446 NPS samples in post-vaccination CSS-1, CSS-2, and CSS-3, respectively. Table 2 provides a summary of the number of samples collected per study CSS and the median age and sex of sampled individuals in each CSS. For CSS-3, children in the “young children” group were older than in the previous CSS (median 4 versus 3 y, respectively, *p*<0.001), and adults from the control communities were younger than those in the vaccinated communities (median 26 versus 39 y, respectively, *p*<0.001).

**Table 2:** Age and sex of individuals studied in vaccinated and control villages in each of the CSSs.

Age Groups	Pre-Vaccination Survey (*n* = 2,094)			Post-Vaccination Surveys									Comparison of the Four Surveys	
				CSS-1 (*n* = 1,168)			CSS-2 (*n* = 1,210)			CSS-3 (*n* = 446)			Control Villages	Vaccinated Villages
	Control Villages (*n* = 948)	Vaccinated Villages (*n* = 1146)	*p*-Value	Control Villages (*n* = 565)	Vaccinated Villages (*n* = 603)	*p*-Value	Control Villages (*n* = 622)	Vaccinated Villages (*n* = 588)	*p*-Valuee	Control Villages (*n* = 212)	Vaccinated Villages (*n* = 234)	*p*-Value	*p*-Value	*p*-Value
**Young children**	*n* = 106	*n* = 113		*n* = 59	*n* = 90		*n* = 70	*n* = 69		*n* = 30	*n* = 23			
Median age (IQR)	3 (2–4)	3 (2–4)	0.434	3 (2–4)	3 (2–4)	0.886	3 (3–4)	3 (2–4)	0.009	4 (4–4)	4 (4–4)	0.946	<0.001	<0.001
Sex (percent male)	57%	51%	0.498	47%	54%	0.503	43%	54%	0.236	45%	57%	0.464	0.282	0.949
**Older children**	*n* = 314	*n* = 418		*n* = 208	*n* = 204		*n* = 219	*n* = 203		*n* = 72	*n* = 91			
Median age (IQR)	9 (7–12)	9 (7–12)	0.831	9 (7–12)	9 (7–12)	0.46	9 (7–11)	9 (7–11)	0.229	7 (6–11)	7 (5–10)	0.58	<0.001	<0.001
Sex (percent male)	55%	51%	0.332	56%	52%	0.429	57%	53%	0.38	53%	50%	0.698	0.895	0.969
**Adults**	*n* = 528	*n* = 615		*n* = 298	*n* = 309		*n* = 333	*n* = 316		*n* = 23	*n* = 21			
Median age (IQR)	37 (22–54)	35 (24–51)	0.915	36 (22–49)	38 (25–53)	0.158	37 (23–51)	35 (21–53)	0.817	26 (17–44)	39 (26–50)	0.001	0.07	0.407
Sex (percent male)	47%	42%	0.095	44%	42%	0.566	43%	47%	0.344	45%	46%	1	0.732	0.414

IQR, interquartile range.

### Prevalence of Pneumococcal Carriage

#### Pre-vaccination survey

The overall prevalence of pneumococcal carriage in the pre-vaccination CSS was high (71.1%) and decreased with age. The proportion of VT among pneumococcal carriers also decreased with age. The prevalence of pneumococcal carriage by age group and by future vaccine arm are shown in Table 3. The overall prevalence was similar in both study arms, but a slightly lower prevalence of VT carriage was observed among villages where universal vaccination was later introduced (not statistically significant for any of the comparisons).

**Table 3:** Comparison of the prevalence of pneumococcal carriage of any pneumococcal serotype, VT serotypes, and NVT serotypes between control and vaccinated villages in each CSS.

Serotypes	Survey	Age Group	Prevalence of Carriage		OR (95% CI)	*p*-Value
			Control Villages, Percent (*n/N*)	Vaccinated Villages, Percent (*n/N*)		
**Any type**	**Pre-vaccination**	2–<5 y	93.4% (99/106)	86.7% (98/113)	0.47 ( 0.14, 1.57)	0.218
		5–<15 y	86.3% (271/314)	82.8% (346/418)	0.76 ( 0.49, 1.20)	0.241
		≥15 y	60.6% (320/528)	57.6% (354/615)	0.89 ( 0.64, 1.26)	0.518
	**CSS-1**	2–<5 y	89.8% (53/59)	87.8% (79/90)	0.83 ( 0.27, 2.52)	0.737
		5–<15 y	63.0% (131/208)	61.3% (125/204)	0.96 ( 0.60, 1.53)	0.872
		≥15 y	24.2% (72/298)	31.1% (96/309)	1.41 ( 0.99, 2.02)	0.058
	**CSS-2**	2–<5 y	64.3% (45/70)	58.0% (40/69)	0.77 ( 0.39, 1.52)	0.445
		5–<15 y	52.5% (115/219)	41.4% (84/203)	0.64 ( 0.37, 1.10)	0.107
		≥15 y	29.1% (97/333)	23.1% (73/316)	0.76 ( 0.49, 1.19)	0.230
	**CSS-3**	2–<5 y	78.9% (30/38)	76.7% (23/30)	0.71 ( 0.11, 4.49)	0.717
		5–<15 y	66.7% (72/108)	68.9% (91/132)	1.16 ( 0.62, 2.18)	0.645
		≥15 y	34.8% (23/66)	29.2% (21/72)	0.77 ( 0.35, 1.70)	0.519
**VT**	**Pre-vaccination**	2–<5 y	53.8% (57/106)	50.4% (57/113)	0.88 ( 0.51, 1.52)	0.640
		5–<15 y	34.7% (109/314)	28.0% (117/418)	0.72 ( 0.47, 1.11)	0.138
		≥15 y	16.7% (88/528)	15.9% (98/615)	0.95 ( 0.69, 1.30)	0.738
	**CSS-1**	2–<5 y	28.8% (17/59)	20.0% (18/90)	0.61 ( 0.25, 1.48)	0.277
		5–<15 y	8.7% (18/208)	5.9% (12/204)	0.67 ( 0.30, 1.47)	0.317
		≥15 y	4.4% (13/298)	4.2% (13/309)	0.96 ( 0.44, 2.11)	0.925
	**CSS-2**	2–<5 y	27.1% (19/70)	21.7% (15/69)	0.88 ( 0.32, 2.42)	0.801
		5–<15 y	8.7% (19/219)	1.5% (3/203)	0.15 ( 0.04, 0.57)	0.005
		≥15 y	3.9% (13/333)	1.3% (4/316)	0.32 ( 0.10, 0.98)	0.046
	**CSS-3**	2–<5 y	23.7% (9/38)	13.3% (4/30)	0.50 ( 0.14, 1.80)	0.287
		5–<15 y	14.8% (16/108)	6.1% (8/132)	0.37 ( 0.15, 0.90)	0.029
		≥15 y	7.6% (5/66)	0.0% (0/72)	0.00 —	—
**NVT**	**Pre-vaccination**	2–<5 y	50.9% (54/106)	54.0% (61/113)	1.16 ( 0.62, 2.16)	0.643
		5–<15 y	58.9% (185/314)	60.8% (254/418)	1.08 ( 0.80, 1.46)	0.613
		≥15 y	41.7% (220/528)	41.0% (252/615)	0.97 ( 0.76, 1.24)	0.815
	**CSS-1**	2–<5 y	66.1% (39/59)	67.8% (61/90)	1.09 ( 0.52, 2.26)	0.823
		5–<15 y	56.3% (117/208)	57.4% (117/204)	1.05 ( 0.71, 1.54)	0.821
		≥15 y	20.5% (61/298)	27.8% (86/309)	1.50 ( 1.03, 2.18)	0.035
	**CSS-2**	2–<5 y	40.0% (28/70)	42.0% (29/69)	1.09 ( 0.55, 2.14)	0.808
		5–<15 y	44.7% (98/219)	40.4% (82/203)	0.86 ( 0.50, 1.47)	0.587
		≥15 y	25.8% (86/333)	22.2% (70/316)	0.84 ( 0.54, 1.30)	0.434
	**CSS-3**	2–<5 y	60.5% (23/38)	63.3% (19/30)	1.02 ( 0.24, 4.26)	0.982
		5–<15 y	52.8% (57/108)	62.9% (83/132)	1.66 ( 0.86, 3.19)	0.129
		≥15 y	30.3% (20/66)	29.2% (21/72)	0.96 ( 0.43, 2.13)	0.924

Surveys were pre-vaccination and 4–6 mo (CSS-1), 12 mo (CSS-2), and 22 mo (CSS-3) post-vaccination.

#### Comparisons of vaccinated and control villages in each of the post-vaccination surveys

**Figure 2:**
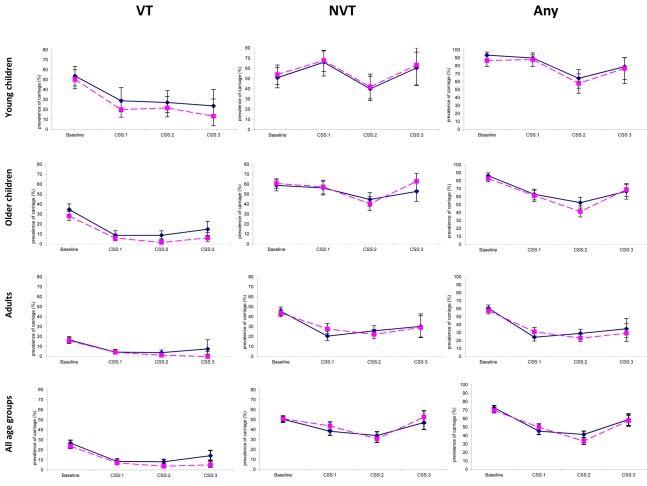
Prevalence of pneumococcal carriage of VT, NVT, and any serotype, in CSSs before and after vaccination of infants and young children, stratified by age group and study arm. Solid lines indicate control villages; dashed lines indicate vaccinated villages.

The overall prevalence of pneumococcal carriage was similar in all post-vaccination CSSs between vaccinated and control villages (Table 3; Figure 2).

The prevalence of carriage of VT pneumococci was lower in vaccinated than in control villages in all post-vaccination CSSs for all age groups, reaching statistical significance in CSS-2 and CSS-3 (except for younger children) (Table 3; Figure 2).

The prevalence of carriage of NVT pneumococci was similar in vaccinated and in control villages in all post-vaccination surveys, except for a higher prevalence of NVT pneumococci among adults in vaccinated communities during CSS-1 (odds ratio [OR] = 1.50 [95% CI 1.03–2.18], *p* = 0.035) (Table 3; Figure 2).

### Time Trends within Trial Arms

The overall prevalence of pneumococcal carriage fell markedly after vaccination and reached minimum levels in post-vaccination CSS-2 in both study arms (Table 4; Figure 2) and in all age groups, except adults in control villages, whose prevalence was lower in CSS-1.

**Table 4:** Comparison of the prevalence of pneumococcal nasopharyngeal carriage of VT serotypes, NVT serotypes, and any pneumococcal serotype between pre-vaccination CSS and each of the post-vaccination CSSs in vaccinated and control villages.

Serotype	Age Group	Control Villages						Vaccinated Villages					
		CSS-1 vs. Baseline		CSS-2 vs. Baseline		CSS-3 vs. Baseline		CSS-1 vs. Baseline		CSS-2 vs. Baseline		CSS-3 vs. Baseline	
		OR (95% CI)	*p*-Value	OR (95% CI)	*p*-Value	OR (95% CI)	*p*-Value	OR (95% CI)	*p*-Value	OR (95% CI)	*p*-Value	OR (95% CI)	*p*-Value
**VT**	2 y to <5 y	0.35 (0.18, 0.70)	0.003	0.32 (0.17, 0.63)	0.001	0.28 (0.11, 0.70)	0.006	0.24 (0.13, 0.46)	<0.001	0.26 (0.13, 0.53)	<0.001	0.18 (0.06, 0.57)	0.004
	5 y to <15 y	0.17 (0.10, 0.29)	<0.001	0.16 (0.10, 0.28)	<0.001	0.25 (0.14, 0.46)	<0.001	0.16 (0.08, 0.29)	<0.001	0.04 (0.01, 0.12)	<0.001	0.15 (0.07, 0.32)	<0.001
	≥15 y	0.23 (0.12, 0.42)	<0.001	0.20 (0.11, 0.37)	<0.001	0.43 (0.17, 1.10)	0.080	0.23 (0.13, 0.42)	<0.001	0.07 (0.02, 0.18)	<0.001	0.00 (—)	—a
	All ages	0.24 (0.17, 0.33)	<0.001	0.23 (0.16, 0.32)	<0.001	0.34 (0.23, 0.53)	<0.001	0.23 (0.16, 0.33)	<0.001	0.12 (0.07, 0.19)	<0.001	0.14 (.08, 0.25)	<0.001
**NVT**	2 y to <5 y	1.19 (0.97, 3.65)	0.061	0.60 (0.32, 1.12)	0.112	1.24 (0.55, 2.76)	0.603	1.78 (1.00, 3.17)	0.051	0.62 (0.34, 1.13)	0.120	1.38 (0.58, 3.28)	0.460
	5 y to <15 y	0.9 (0.63, 1.12)	0.581	0.56 (0.39, 0.80)	0.001	0.74 (0.48, 1.16)	0.190	0.86 (0.61, 1.22)	0.407	0.43 (0.30, 0.61)	<0.001	1.00 (0.66, 1.51)	0.998
	≥15 y	0.30 (0.21, 0.41)	<0.001	0.41 (0.30, 0.56)	<0.001	0.46 (0.26, 0.82)	0.008	0.50 (0.37, 0.67)	<0.001	0.35 (0.25, 0.47)	<0.001	0.53 (0.31, 0.92)	0.024
	All ages	0.58 (0.47, 0.72)	<0.001	0.48 (0.39, 0.60)	<0.001	0.68 (0.50, 0.93)	0.015	0.72 (0.59, 0.89)	0.002	0.39 (0.32, 0.49)	<0.001	0.88 (0.65, 1.18)	0.393
**Any type**	2 y to <5 y	0.67 (0.21, 2.12)	0.498	0.11 (0.04, 0.29)	<0.001	0.23 (0.07, 0.77)	0.018	1.15 (0.49, 2.68)	0.746	0.21 (0.10, 0.43)	<0.001	0.54 (0.18, 1.58)	0.260
	5 y to <15 y	0.27 (0.17, 0.41)	<0.001	0.16 (0.11, 0.25)	<0.001	0.26 (0.15, 0.45)	<0.001	0.31 (0.21, 0.46)	<0.001	0.13 (0.09, 0.20)	<0.001	0.38 (0.25, 0.60)	<0.001
	≥15 y	0.20 (0.14, 0.28)	<0.001	0.26 (0.19, 0.35)	<0.001	0.32 (0.19, 0.56)	<0.001	0.32 (0.24, 0.44)	<0.001	0.20 (0.15, 0.28)	<0.001	0.30 (0.17, 0.51)	<0.001
	All ages	0.26 (0.20, 0.33)	<0.001	0.22 (0.17, 0.27)	<0.001	0.34 (0.24, 0.47)	<0.001	0.37 (0.30, 0.46)	<0.001	0.17 (0.14, 0.22)	<0.001	0.41 (0.30, 0.56)	<0.001

Estimates were made using a mixed effects logistic regression model combining data from all CSSs, adjusted by age (within each age group), with village included as a random effect. VT includes the seven serotypes included in the vaccine and serotype 6A. NVT includes all other pneumococcal serotypes.

a Paired sign test used to compare prevalence in study CSS, as the OR = 0 and could not be tested using logistic regression.

The decrease in the prevalence of VT carriage seen in both vaccinated and control villages was statistically significant for all age groups and any post-vaccination CSS when compared to the pre-vaccination survey, with one exception in the control group (see Table 4; Figure 2).

For NVT pneumococci, changes in the pattern of carriage differed according to study age group (Table 4; Figure 2). Among the youngest children, there was an increase in prevalence of NVT pneumococci between baseline and CSS-1 that returned to baseline levels in CSS-2 and CSS-3. Among older children, there was a decrease in carriage of NVT pneumococci in CSS-2 in both study arms, but carriage increased in CSS-3 to its former level. Among adults, there was a significant decrease in carriage of NVT between baseline and post-vaccination CSS-1 in both study arms, and the final prevalence in CSS-3 was still lower than baseline (OR = 0.46 [95% CI 0.26–0.82], *p* = 0.008, and OR = 0.53 [95% CI 0.31–0.92], *p* = 0.024, for control and vaccinated villages, respectively).

In the time trend analysis, the overall decrease between the pre-vaccination CSS and CSS-1 was bigger for VT than for NVT in both vaccinated (OR = 0.24 [95% CI 0.17–0.33] for VT and OR = 0.58 [95% CI 0.47–0.72] for NVT) and control villages (OR = 0.23 [95% CI 0.16–0.33] for VT and OR = 0.72 [95% CI 0.59–0.89] for NVT) (Table 4).

### Individual Serotypes

The most prevalent serotypes in the pre-vaccination CSS among VT pneumococci were serotypes 23F (*n* = 86), 6A (*n* = 84), and 6B (*n* = 80), and among NVT pneumococci, serotypes 3 (*n* = 182), 11 (*n* = 81), and 7C (*n* = 52). During the post-vaccination CSSs, serotype 3 remained the most prevalent among NVT isolates, followed by serotype 11, except for CSS-3, when serotypes 11 and 19A were the most common. The most prevalent VT serotypes in the post-vaccination surveys were serotypes 19F and 6A. Table 5 shows the prevalence of VT serotypes and the most prevalent NVT serotypes for each CSS in vaccinated and control villages. The prevalence of carriage of serotype 5 pneumococci increased in vaccinated villages from 0% to 4.7%. Seven of the 11 serotype 5 isolates detected in CSS-3, were obtained from older children (64%), and seven came from the same village.

**Table 5:** Pneumococcal carriage of individual serotypes in the pre-vaccination and post-vaccination surveys in control and vaccinated villages.

Individual Serotypes	Pre-Vaccination CSS			Post-Vaccination Surveys								
				CSS-1			CSS-2			CSS-3		
	Control Villages (*n* = 948), *n*(%)	Vaccinated Villages (*n* = 1,146), *n* (%)	OR (95% CI)	Control Villages (*n* = 565), *n* (%)	Vaccinated Villages (*n* = 603), *n* (%)	OR (95% CI)	Control Villages (*n* = 622), *n* (%)	Vaccinated Villages (*n* = 588), *n* (%)	OR (95% CI)	Control Villages (*n* = 212), *n* (%)	Vaccinated Villages (*n* = 234), *n* (%)	OR (95% CI)
**VT**												
4	25(2.6)	49(4.3)	1.65(0.69, 3.91)	3(0.5)	0(0.0)	NC	1(0.2)	0(0.0)	NC	3(1.4)	0(0.0)	NC
6B	40(4.2)	40(3.5)	0.82(0.47, 1.43)	7(1.2)	8(1.3)	1.07(0.49, 2.37)	10(1.6)	6(1.0)	0.63(0.13, 3.02)	5(2.4)	1(0.4)	0.18(0.02, 1.51)
9V	40(4.3)	29(2.5)	0.57(0.29, 1.12)	1(0.2%)	4(0.7)	3.77(0.33, 42.52)	3(0.5)	0(0.0)	—	1(0.5)	2(0.9)	1.82(0.15, 22.70)
14	21(2.2)	15(1.3)	0.59(0.20, 1.76)	7(1.2%)	2(0.3)	0.27(0.03, 2.32)	1(0.2)	1(0.2)	1.06(0.07, 15.77)	1(0.5)	0(0.0)	—
18C	30(3.2)	24(2.1)	0.65(0.30, 1.41)	2(0.4%)	1(0.2)	0.47(0.04, 4.89)	4(0.6)	0(0.0)	—	5(2.4)	0(0.0)	—
19F	33(3.5)	37(3.2)	0.93(0.46, 1.84)	12(2.1)	8(1.3)	0.62(0.18, 2.13)	8(1.3)	5(0.9)	0.66(0.16, 2.79)	3(1.4)	5(2.1)	1.52(0.40, 5.76)
23F	52(5.5)	34(3.0)	0.53(0.28, 1.01)	7(1.2)	6(1.0)	0.80(0.26, 2.49)	11(1.8)	5(0.9)	0.48(0.17, 1.31)	7(3.3)	0(0.0)	—
6A	26(2.7)	58(5.1)	1.89(1.00, 3.57)	9(1.6)	16(2.7)	1.68(0.76, 3.72)	13(2.1)	6(1.0)	0.48(0.16, 1.44)	6(2.8)	4(1.7)	0.60(0.18, 1.96)
**NVT** a												
1	4(0.4)	4(0.3)	0.83(0.12, 5.59)	3(0.5)	0(0.0)	—	4(0.6)	4(0.7)	1.06(0.32, 3.50)	0(0.0)	2(0.9)	—
5	6(0.6)	5(0.1)	0.14(0.01, 1.31)	5(0.9)	1(0.2)	0.19(0.02, 1.98)	0(0.0)	0(0.0)	—	0(0.0)	11(4.7)	—b
3	76(8.0)	106(9.2)	1.17(0.76, 1.81)	27(4.8)	19(3.2)	0.65(0.30, 1.38)	36(5.8)	14(2.4)	0.40(0.22, 0.73)	8(3.8)	8(3.4)	NC
7C	10(1.1)	42(3.7)	3.57(1.33, 9.54)	2(0.4)	0(0.0)	NC	2(0.3)	2(0.3)	NC	1(0.5)	0(0.0)	NC
11	37(3.9)	44(3.8)	0.98(0.50, 1.95)	10(1.8)	20(3.3)	1.90(0.68, 5.33)	17(2.7)	13(2.2)	0.80 (0.27, 2.36)	9(4.2)	13(5.6)	NC
15B	15(1.6)	23(2.0)	1.27(0.37, 4.35)	7(1.2)	10(1.7)	NC	9(1.4)	4(0.7)	NC	7(3.3)	5(2.1)	NC
19A	27(2.8)	20(1.7)	0.61(0.30, 1.22)	3 (0.5)	8 (1.3)	2.52(0.85, 7.44)	11(1.8)	3(0.7)	0.28(0.06, 1.47)	6(2.8)	15(6.4)	2.35^c^ (0.73, 7.62)
23B	8(0.8)	2(0.2)	0.21(0.04, 0.98)	6(1.1)	2(0.3)	0.31(0.05, 1.88)	0(0)	3(0.5)	—	1(0.5)	8(3.4)	7.47^c^ (1.09, 51.16)
34	14(1.5)	30(2.6)	1.79(0.75, 4.31)	15(2.7)	21(3.5)	1.32(0.34, 5.11)	11(1.8)	9(1.5)	NC	8(3.8)	3(1.3)	NC

VT includes the seven serotypes included in the vaccine and serotype 6A. NVT includes all other pneumococcal serotypes. All age groups are combined in this table.

a Only those serotypes that were isolated in more than 3% of the samples in at least one group have been included. ORs were calculated only for serotypes with at least 15 isolates in one group.

b *p*-Value for this increase when considering risk difference and allowing clustering by village is *p* = 0.151; 63.6% of the isolates (7/11) were detected in the same village.

c The increase of prevalence in the vaccinated group during CSS-3 was led by an increase in young children. From the 30 samples collected in the vaccinated arm among children 2 y to <5 y of age, 16.7% (*n* = 5) were isolate 19A and 10% (*n* = 3) were isolate 23B.

NC, not calculated.

Details of the time trends for individual serotypes within vaccinated and control villages are shown in Table S4. Most of the VT serotypes significantly decreased compared to the baseline CSS in vaccinated and control villages. Decrease of serotype 6A was less prominent in control villages. Of the most prevalent NVT serotypes, serotype 3 and 7C decreased in vaccinated communities, and serotypes 5, 19A, and 23B significantly increased in vaccinated communities in CSS-3.

## Discussion

To anticipate the potential long-term effects of the introduction of pneumococcal conjugate vaccination in sub-Saharan Africa, we have conducted a novel randomized, controlled trial in which a population of pneumococci was exposed to maximum immune pressure by vaccinating all residents of 11 Gambian villages with PCV-7. We measured the prevalence of pneumococcal carriage in these villages before and on three occasions after vaccination, and compared these findings with those obtained in control villages where only young children had been vaccinated with PCV-7. Time trends were investigated by comparing pneumococcal carriage within both groups of villages before and after vaccination.

By the time this study was ready to start, the results of the Gambian PCV-9 trial had become available [20], showing a high level of protection against IPD in children under the age of 30 mo, so it was considered unethical to proceed with a trial in which all children in this age group did not receive PCV, as had been originally intended. This design modification weakened the ability of the study to show the direct effect of vaccination on the community through differences between vaccinated and control villages, but serendipitously allowed demonstration of a potential herd effect resulting from immunization of children in control communities. Changes in the prevalence of carriage between the before and after vaccination surveys need to be interpreted with caution because of the potential influence of temporal trends unrelated to vaccination. However, methods used for sampling and isolation of pneumococci were consistent over the study period, including pre- and post-vaccination surveys. Furthermore, the investigators are not aware of any changes in the study villages that could have affected risk factors for carriage during the conduct of the study, apart from the administration of azithromycin at the time of the last CSS, an event which has been addressed in the analysis.

A time trend analysis showed a marked drop in the prevalence of carriage of VT pneumococci among all age groups in vaccinated and control villages following vaccination. The decline in the prevalence of VT carriage in older children and adults in the control villages strongly suggests a herd effect resulting from vaccination of infants and toddlers in those villages. This effect was apparent as early as 6 mo after vaccination and persisted for at least the next 2 y. This finding is in line with results from a pre-vaccination observational study conducted in the same villages that suggested that transmission occurred mainly from young children to older members of the family [26]. Although the study villages were separated by at least 3 km, some mingling between communities may have contributed to the herd effect observed in the control villages, as some older children resident in these villages attended school in neighboring villages. Because of the high level of transmission of pneumococcal infection in Africa [26], there have been concerns that induction of herd immunity through vaccination of infants might be more difficult to achieve in this continent than elsewhere. We have shown here, to our knowledge for the first time in Africa, a herd effect on carriage of VT pneumococci, which might be translated into herd protection against IPD in adults, following routine immunization of infants and young children, as has been observed in the US [4]. Our findings are relevant for other countries in Africa contemplating the introduction of PCVs where the pattern of pneumococcal infection is similar to that in The Gambia. Whether a similar effect will be observed in countries with a high prevalence of HIV (HIV-infected adults are more susceptible to being carriers of pediatric pneumococcal serotypes) remains unknown.

The prevalence of carriage of VT pneumococci was slightly lower, but statistically significantly so, in individuals from vaccinated than from control villages in most post-vaccination comparisons. Thus, vaccination of older children and adults with PCV-7 had some additional effect in decreasing rates of carriage of VT pneumococci, but this effect was not marked.

The prevalence of carriage of NVT pneumococci among adults was lower in the post-vaccination surveys than in the pre-vaccination survey in both control and vaccinated communities. This decrease was not observed in other age groups in either vaccinated or control villages, so this may have been a chance finding. There was a significant gap between the time that the pre-vaccination study was done and the first post-vaccination survey, and it is possible that there may have been some change in the pattern of carriage in the study villages during this time. However, surveys undertaken in The Gambia over a period of many years, including several conducted in the study area, have consistently shown very high levels of carriage, and it is unlikely that the overall level of carriage would have changed during this period, although there could have been some changes in serotype distribution [7],[23],[24],[26],[28],[33]. “Up and down” trends in the prevalence of different serotypes have been documented previously in the area over time [26]. Another possible explanation includes a nonspecific effect on health produced by the intervention, but such an effect should have been observed in all age groups. Still, the observed decrease of NVT pneumococci prevalence from the pre-vaccination CSS to post-vaccination CSS-1 was significantly lower than the decrease of VT pneumococci prevalence observed for these two CSSs in both vaccinated and control communities.

The absence of a significant increase in the prevalence of carriage of NVT pneumococci in any age group, and the limited differences between control and vaccinated villages, are surprising, as many previous studies [8],[9],[12], including some conducted in The Gambia [7],[34], have shown a marked increase in the prevalence of carriage of NVT pneumococci after vaccination. These previous carriage studies conducted in The Gambia focused on infants and younger children [7],[34], an age group not sampled in our study. Lack of an increase in NVT pneumococci prevalence has previously been documented in a carriage study of household contacts among Native Americans whose children participated in a PCV-7 vaccine trial [35]. The investigators observed no increase in carriage of NVT pneumococci among older children and adults within households of children vaccinated with PCV-7, although a reduction in carriage of VT pneumococci was found in the adult age group. An increase in carriage of NVT pneumococci was found only in non-vaccinated household members <5 y of age from households with PCV-7 vaccinees. Encouragingly, vaccination of older children and adults in our study did not add selection pressure towards an overall increase in carriage of NVT pneumococci.

We have also observed in our study a decrease of the overall pneumococcal carriage in the post-vaccination CSSs compared to the pre-vaccination CSS. Because there is little serotype replacement, PCV introduction might also have had an effect on the overall prevalence of pneumococcal carriage at least up to 2 y after vaccination. Pneumococcal prevalence reached its lowest levels in the post-vaccination CSS-2. As mentioned above, secular trends or the health effect of the intervention could partly explain such results.

An increase in carriage of serotype 5 pneumococci seen in vaccinated villages in CSS-3 was due largely to an outbreak in one village and may have been a chance finding unrelated to vaccination, an event similar to one observed in the study community before any intervention was performed [26]. However, the possibility of an association with PCV vaccination is of concern as this serotype, along with serotype 1, is a major cause of IPD in The Gambia [36] and elsewhere in Africa [15],[22],[27]. Both serotype 5 and serotype 1 pneumococci also have the potential to cause epidemics [37]–[39]. An increase in the prevalence of carriage of NVT serotypes such as 19A and 23B was observed during CSS-3 in the vaccinated villages (shown in both the arm comparison and the time trend analysis), probably driven by young children. An increase in carriage of serotype 19A after PCV vaccination has been documented previously in The Gambia [7] and elsewhere [8],[9],[11],[12]. An increase has also been widely documented for IPD caused by this serotype [4],[10],[11], demonstrating lack of cross-protection of PCV-7 for serotype 19A. Serotype 6A was still present in all post-vaccination surveys but with lower prevalence in each of the post-vaccination CSSs compared to the pre-vaccination survey in both vaccinated and control communities, suggesting the expected vaccine protective effect in carriage, and potentially on IPD, a finding that justifies inclusion of this serotype in the VT group. However, the 6A decrease was statistically significant only among vaccinated communities. Serotype 3 was the most prevalent NVT serotype detected both before and after vaccination in this study, and serotype 19A was shown to be the most common at some point after vaccination. Both of these serotypes and serotype 6A are contained in the PCV-13, which has been shown to be immunogenic in infants, with potential protective effect against serotype-specific IPD [40]–[42]. PCV-13 has recently replaced PCV-7 in routine childhood immunization programs in The Gambia and would be expected to provide coverage of these serotypes.

This study faced an additional challenge. The last post-vaccination survey was disrupted by the administration of azithromycin to individuals in several study villages as part of a national trachoma elimination program that had not been envisaged when the carriage study was planned. To avoid any direct or indirect effect resulting from this treatment, CSS-3 analyses include only a third of the target number of samples, all collected before treatment started in the area, and therefore, this survey lacked statistical power for comparison of vaccinated versus control villages.

Despite the challenges and limitations of this study, the main findings are robust and very encouraging. Vaccination of young Gambian children reduced carriage of VT pneumococci in vaccinated children but also in vaccinated and non-vaccinated older children and adults, revealing to our knowledge for the first time in Africa a potential herd effect from vaccination of young children. The immunological pressure induced by vaccinating whole communities did not lead to a community-wide increase in carriage of NVT pneumococci during a 2-y period after vaccination. Further long-term follow-up carriage studies in this community are planned.

## Supporting Information

Table S1 **Characteristics of individuals included in the pre-vaccination and post-vaccination CSSs.** Only information from individuals above 15 y of age are included (data were not available for younger individuals in some of the CSSs).
(DOCX)

Table S2 **Vaccination status of village residents at the midpoint of each post-vaccination survey.** Age groups are defined by the age of individuals at the midpoint of the CSS.
(DOCX)

Table S3 **Time (days) from administration of the first dose of vaccine in each of the villages to the collection of the NPSs included in each of the post-vaccination CSSs in control and vaccinated villages.**
(DOCX)

Table S4 **Comparison of the prevalence of pneumococcal carriage of individual serotypes in each post-vaccination CSS compared to the pre-vaccination CSS in control and vaccinated villages.** All age groups have been combined in this table.
(DOCX)

Text S1 **Trial protocol.**
(PDF)

Text S2 **CONSORT checklist.**
(DOC)

## Abbreviations

CSS: cross-sectional survey
IPD: invasive pneumococcal disease
MRC: Medical Research Council
NPS: nasopharyngeal swab
NVT: non-vaccine type
OR: odds ratio
PCV: pneumococcal conjugate vaccine
VT: vaccine type
