# Supplementary material for: Effects of Community-Wide Vaccination with PCV-7 on Pneumococcal Nasopharyngeal Carriage in The Gambia: A Cluster-Randomized Trial
Source: PLoS Med. 2011 Oct 18;8(10):e1001107. doi: 10.1371/journal.pmed.1001107 (PMC3196470; doi:10.1371/journal.pmed.1001107)
Supplement: Table S1 — Characteristics of individuals included in the pre-vaccination and post-vaccination CSSs. Only information from individuals above 15 y of age are included (data were not available for younger individuals in some of the CSSs). (DOCX) [file pmed.1001107.s001.docx]

| Variable | | Pre-vaccination | | | | Post-vaccination | | | | | | | | | | | |
| --- | --- | --- | --- | --- | --- | --- | --- | --- | --- | --- | --- | --- | --- | --- | --- | --- | --- |
|  |  |  |  |  |  | CSS1 | | | | CSS2 | | | | CSS3 | | | |
|  |  | Control  Villages | | Vaccinated  Villages | | Control  Villages | | Vaccinated  Villages | | Control Villages | | Vaccinated Villages | | Control Villages | | Vaccinated Villages | |
| Ethnic group |  | n | % | n | % | n | % | n | % | n | % | n | % | n | % | n | % |
|  | Mandinka | 4 | 1% | 198 | 33% | 0 | 0% | 113 | 36% | 1 | 0% | 116 | 37% | 1 | 2% | 28 | 39% |
|  | Jola | 492 | 94% | 357 | 59% | 283 | 95% | 179 | 58% | 311 | 93% | 185 | 58% | 60 | 91% | 42 | 58% |
|  | Fula | 16 | 3% | 30 | 5% | 9 | 3% | 12 | 4% | 11 | 3% | 9 | 3% | 3 | 5% | 0 | 0% |
|  | Other | 12 | 2% | 22 | 4% | 6 | 2% | 6 | 2% | 10 | 3% | 7 | 2% | 2 | 3% | 2 | 3% |
|  | *Total* | *524* |  | *607* |  | *298* |  | *310* |  | *333* |  | *317* |  | *66* |  | *72* |  |
| Occupation | Business | 9 | 2% | 13 | 2% | 12 | 4% | 2 | 1% | 3 | 1% | 4 | 1% | 0 | - | 1 | 1% |
|  | Farmer/Agricultural | 332 | 63% | 372 | 61% | 132 | 44% | 196 | 63% | 214 | 64% | 202 | 64% | 31 | 47% | 46 | 64% |
|  | Housewife | 27 | 5% | 37 | 6% | 47 | 16% | 32 | 10% | 13 | 4% | 11 | 3% | 12 | 18% | 11 | 15% |
|  | Student | 91 | 17% | 84 | 14% | 59 | 20% | 47 | 15% | 67 | 20% | 57 | 18% | 18 | 27% | 4 | 6% |
|  | Teacher | 12 | 2% | 16 | 3% | 6 | 2% | 3 | 1% | 1 | <1% | 3 | 1% | 1 | 2% | 3 | 4% |
|  | Other | 53 | 10% | 85 | 14% | 41 | 14% | 29 | 10% | 35 | 11% | 40 | 13% | 4 | 6% | 7 | 10% |
|  | *Total* | *524* |  | *607* |  | *297* |  | *309* |  | *333* |  | *317* |  | *66* |  | *72* |  |
| Years of schooling | Less than 1 y | 318 | 62% | 345 | 58% | 158 | 53% | 162 | 53% | 179 | 54% | 148 | 47% | 27 | 41% | 28 | 39% |
|  | 1-6 years | 86 | 17% | 118 | 20% | 58 | 20% | 49 | 16% | 49 | 15% | 61 | 19% | 18 | 27% | 20 | 28% |
|  | 7-10 years | 91 | 18% | 91 | 15% | 55 | 19% | 59 | 19% | 70 | 21% | 64 | 20% | 18 | 27% | 10 | 14% |
|  | More than 10 y | 22 | 4% | 45 | 8% | 26 | 9% | 36 | 12% | 34 | 10% | 44 | 14% | 3 | 5% | 14 | 19% |
|  | *Total* | 517 |  | 599 |  | 297 |  | 306 |  | 332 |  | 317 |  | 66 |  | 72 |  |
