# Supplementary material for: Effects of Community-Wide Vaccination with PCV-7 on Pneumococcal Nasopharyngeal Carriage in The Gambia: A Cluster-Randomized Trial
Source: PLoS Med. 2011 Oct 18;8(10):e1001107. doi: 10.1371/journal.pmed.1001107 (PMC3196470; doi:10.1371/journal.pmed.1001107)
Supplement: Table S2 — Vaccination status of village residents at the midpoint of each post-vaccination survey. Age groups are defined by the age of individuals at the midpoint of the CSS. (DOCX) [file pmed.1001107.s002.docx]

| Vaccination Status | CSS1  (Mid point 23^rd^ Jan 2007) | | CSS2  (Mid point 15 Aug 2007) | | CSS3  (Mid point 3^rd^ Jun 2008) | |
| --- | --- | --- | --- | --- | --- | --- |
|  | Control villages  n (%) | Vaccinated villages  n (%) | Control villages  n (%) | Vaccinated villages  n (%) | Control villages  n (%) | Vaccinated villages  n (%) |
| At the mid-point of the cross-sectional survey:   1. Children <2 years    - 1. Non-vaccinated      2. ≥ 1 dose PCV-7      3. 1 dose meningococcal 2. Children 2-<5 years    - 1. Non-vaccinated      2. ≥ 1 dose PCV-7      3. 1 dose meningococcal 3. Children 5-<15 years    - 1. Non-vaccinated      2. ≥ 1 dose PCV-7      3. 1 dose meningococcal 4. Adults >15 years    - 1. Non-vaccinated      2. ≥ 1 dose PCV-7      3. 1 dose meningococcal 5. All ages    - 1. Non-vaccinated      2. ≥ 1 dose PCV-7      3. 1 dose meningococcal | 76 (40%)  113 (60%)  0 (0%)  139 (37%)  73 (19%)  164 (43%)  342 (34%)  1 (0%)  664 (66%)  1119 (52%)  1 (0%)  1020 (48%)  1676 (45%)  188 (5%)  1848 (50%) | 52 (24%)  165 (76%)  0 (0%)  44 (13%)  307 (87%)  0 (0%)  118 (12%)  870 (88%)  0 (0%)  310 (16%)  1620 (84%)  0 (0%)  524 (15%)  2962 (85%)  0 (0%) | 28 (16%)  149 (84%)  0 (0%)  25 (10%)  103 (39%)  135 (51%)  127 (15%)  2 (0%)  724 (85%)  511 (28%)  0 (0%)  1286 (72%)  691 (22%)  254 (8%)  2145 (69%) | 23 (11%)  188 (89%)  0 (0%)  13 (4%)  288 (96%)  0 (0%)  50 (6%)  853 (94%)  0 (0%)  177 (10%)  1601 (90%)  0 (0%)  263 (8%)  2930 (92%)  0 (0%) | 58 (33%)  119 (67%)  1 (0%)  36 (13%)  170 (62%)  67 (25%)  132 (15%)  2 (0%)  743 (85%)  539 (28%)  0 (0%)  1399 (72%)  765 (23%)  291 (9%)  2210 (68%) | 58 (27%)  153 (73%)  0 (0%)  41 (13%)  282 (87%)  0 (0%)  79 (8%)  856 (92%)  0 (0%)  264 (13%)  1705 (87%)  0 (0%)  442 (13%)  2996 (87%)  0 (0%) |
