# Supplementary material for: Effects of Community-Wide Vaccination with PCV-7 on Pneumococcal Nasopharyngeal Carriage in The Gambia: A Cluster-Randomized Trial
Source: PLoS Med. 2011 Oct 18;8(10):e1001107. doi: 10.1371/journal.pmed.1001107 (PMC3196470; doi:10.1371/journal.pmed.1001107)
Supplement: Table S3 — Time (days) from administration of the first dose of vaccine in each of the villages to the collection of the NPSs included in each of the post-vaccination CSSs in control and vaccinated villages. (DOCX) [file pmed.1001107.s003.docx]

| Cross-sectional survey | Control Villages  Median  (Q25-Q75) | Vaccinated Villages  Median  (Q25-Q75) |
| --- | --- | --- |
| - CSS-1 - CSS-2 - CSS-3 | 177  (158 - 191)  360  (359 – 365)  632  (590 – 641) | 125  (123 – 128)  365  (365 – 370)  671  (626 – 672) |

*Footnote: Vaccination date was considered per each village the day that PCV-7 vaccination started in such village. The first round of vaccination was conducted in 1-4 days per village.*
