# Supplementary material for: Effects of Community-Wide Vaccination with PCV-7 on Pneumococcal Nasopharyngeal Carriage in The Gambia: A Cluster-Randomized Trial
Source: PLoS Med. 2011 Oct 18;8(10):e1001107. doi: 10.1371/journal.pmed.1001107 (PMC3196470; doi:10.1371/journal.pmed.1001107)
Supplement: Table S4 — Comparison of the prevalence of pneumococcal carriage of individual serotypes in each post-vaccination CSS compared to the pre-vaccination CSS in control and vaccinated villages. All age groups have been combined in this table. (DOCX) [file pmed.1001107.s004.docx]

| Serotype  group | Serotype | Control villages | | | | | | Vaccinated villages | | | | | |
| --- | --- | --- | --- | --- | --- | --- | --- | --- | --- | --- | --- | --- | --- |
|  |  | CSS-1 vs Baseline | | CSS-2 vs Baseline | | CSS-3 vs Baseline | | CSS-1 vs Baseline | | CSS-2 vs Baseline | | CSS-3 vs Baseline | |
|  |  | OR  95%CI | p-value | OR  95%CI | p-value | OR  95%CI | p-value | OR  95%CI | p-value | OR  95%CI | p-value | OR  95%CI | p-value |
| VT | 4 | 0.19  (0.04,0.99) | **0.049** | 0.06  (0.01,0.32) | **0.001** | 0.48  (0.10,2.29) | 0.362 | 0.00  --- | **<0.001** | 0.00  --- | **<0.001** | 0.00  --- | **<0.001** |
|  | 6B | 0.28  (0.15,0.50) | **<0.001** | 0.36  (0.16,0.83) | **0.016** | 0.46  (0.17,1.22) | 0.118 | 0.36  (0.20,0.67) | **0.001** | 0.28  (0.07,1.16) | 0.078 | 0.10  (0.01,0.74) | **0.024** |
|  | 9V | 0.04  (0.00,0.38) | **0.005** | 0.10  (0.03,0.41) | **0.001** | 0.09  (0.01,0.79) | **0.030** | 0.24  (0.06,0.93) | **0.039** | 0.00  --- | **<0.001** | 0.25  (0.04,1.67) | 0.153 |
|  | 14 | 0.54  (0.17,0.33) | **<0.001** | 0.07  (0.01,0.50) | **0.008** | 0.17  (0.01,1.89) | 0.150 | 0.25  (0.02,2.69) | 0.250 | 0.13  (0.02,0.65) | **0.013** | 0.00  --- | 0.090 |
|  | 18C | 1.19  (0.12,2.35) | 0.413 | 0.20  (0.07,0.53) | 0.112 | 0.68  (0.12,3.82) | 0.660 | 0.08  (0.01,0.66) | **0.020** | 0.00  --- | **<0.001** | 0.00  --- | **0.024** |
|  | 19F | 0.59  (0.17,2.06) | 0.405 | 0.35  (0.16,0.77) | **0.009** | 0.31  (0.06,1.61) | 0.163 | 0.40  (0.13,1.19) | 0.100 | 0.25  (0.06,1.09) | 0.066 | 0.58  (0.27,1.24) | 0.161 |
|  | 23F | 0.21  (0.11,0.38) | **<0.001** | 0.30  (0.16,0.55) | **<0.001** | 0.45  (0.26,0.79) | **0.005** | 0.32  (0.11,0.93) | **0.036** | 0.27  (0.10,0.73) | **0.010** | 0.00  --- | **0.004** |
|  | 6A | 0.56  (0.23,1.40) | 0.217 | 0.75  (0.23,2.45) | 0.632 | 0.889  (0.38,2.06) | 0.784 | 0.50  (0.25,0.99) | **0.047** | 0.19  (0.08,0.45) | **<0.001** | 0.28  (0.12,0.64) | **0.003** |
| NVT | 1 | 1.23  (0.15,10.09) | 0.847 | 1.50  (0.23,9.83) | 0.674 | 0.00  --- | 1.000 | 0.00  --- | 0.305 | 1.95  (0.46,8.30) | 0.368 | 2.18  (0.38,12.36) | 0.379 |
|  | 3 | 0.56  (0.28,1.12) | 0.100 | 0.69  (0.40,1.21) | 0.197 | 0.38  (0.21,0.69) | **0.002** | 0.31  (0.19,0.51) | **<0.001** | 0.23  (0.13,0.42) | **<0.001** | 0.30  (0.16,0.57) | **<0.001** |
|  | 5 | 1.37  (0.25,7.52) | 0.715 | 0.00  --- | 0.087 | 0.00  --- | 0.599 | 1.90  (0.11,33.84) | 0.662 | 0.00  --- | 1.000 | 55.64  (4.59,673.9) | **0.002** |
|  | 7C | 0.33  (0.11,1.00) | **0.051** | 0.30  (0.07,1.22) | 0.094 | 0.43  (0.04,4.67) | 0.491 | 0.00  --- | **<0.001** | 0.09  (0.03,0.27) | **<0.001** | 0.00  --- | **0.001** |
|  | 11 | 0.44  (0.21,0.89) | **0.023** | 0.68  (0.25,1.86) | 0.459 | 0.96  (0.39,2.40) | 0.935 | 0.85  (0.36,2.03) | 0.713 | 0.56  (0.31,1.03) | **<0.001** | 1.30  (0.54,3.15) | 0.559 |
|  | 13 | 2.01  (0.99,4.05) | 0.052 | 1.21  (0.33,4.43) | 0.774 | 1.56  (0.80,3.04) | 0.192 | 2.64  (0.80,8.71) | 0.111 | 2.27  (0.87,5.89) | 0.092 | 1.59  (0.40,6.41) | 0.511 |
|  | 15B | 0.77  (0.18,3.26) | 0.723 | 0.90  (0.40,2.05) | 0.811 | 1.88  (0.64,5.50) | 0.252 | 0.72  (0.25,2.05) | 0.469 | 0.33  (0.10,1.09) | 0.068 | 0.87  (0.10,7.19) | 0.894 |
|  | 19A | 0.176  (0.07,0.45) | **<0.001** | 0.60  (0.22,1.64) | 0.317 | 0.72  (0.23,2.25) | 0.571 | 1.81  (0.23,14.43) | 0.542 | 0.28  (0.06,1.33) | 0.110 | 2.97  (1.45,6.07) | **0.003** |
|  | 23B | 1.23  (0.24,6.40) | 0.804 | 0.00  --- | **<0.001** | 0.40  (0.05,3.43) | 0.403 | 1.81  (0.23,14.43) | 0.574 | 2.89  (0.67,12.04) | 0.153 | 15.35  (3.11,75.70) | **0.001** |
|  | 34 | 1.78  (0.81,3.94) | 0.152 | 1.18  (0.40,3.52) | 0.765 | 2.02  (0.67,6.08) | 0.209 | 1.33  (0.44,4.00) | 0.616 | 0.57  (0.28,1.19) | 0.138 | 0.42  (0.12,1.49) | 0.179 |
