# Supplementary material for: Effects of Community-Wide Vaccination with PCV-7 on Pneumococcal Nasopharyngeal Carriage in The Gambia: A Cluster-Randomized Trial
Source: PLoS Med. 2011 Oct 18;8(10):e1001107. doi: 10.1371/journal.pmed.1001107 (PMC3196470; doi:10.1371/journal.pmed.1001107)
Supplement: Text S1 — Trial protocol. (PDF) [file pmed.1001107.s005.pdf]

Study Protocol

Community Randomised Controlled Trial to assess impact of vaccination with a Pneumococcal Conjugate Vaccine on pneumococcal nasopharyngeal carriage in The Gambia

SCC number 1032
Ethics number SCC 1032
International Standard Randomised Controlled Trial number:
ISRCTN51695599

Sponsor: MRC Laboratories
PO Box 273, Banjul, The Gambia
Ph 00220 4495 442
Fax 00220 449 7924

Principal Investigator: Prof R A Adegbola
MRC Laboratories
PO Box 273, Banjul, The Gambia
Ph 00220 4495 442 ext 392
Fax 00220 449 7924

Confidential and Proprietary information

The present document is to be used by MRC representatives, investigators, regulatory authorities, institutional review boards and ethics committees. This document is confidential and cannot be communicated to third parties or used for publication or conferences without written approval of the sponsor.

Team Roster

Principal Investigator

Prof Richard Adegbola
MRC, The Gambia
Ext 392
Email: radegbola@mrc.gm

Co-Investigators
Dr Philip Hill (clinician epidemiologist)
Dr Uzochukwu Egere (trial clinician)
Mr Mark Saaka
Kawsu Sankareh
Dr Martin Antonio

External Advisors
Prof Brian Spratt
Prof Brian Greenwood

Field Researchers

Supervised by:
Man-ansu Kinteh, Jereba Darboe

Laboratory Researchers

Supervised by:
Kawsu Sankareh, Mark Saaka

Participating Site
MRC Labs, Fajara, The Gambia

Trial Statistician
Dr Yin Bun Cheung
YinBun.Cheung@lshtm.ac.uk

Data Manager and database designer

George Lahai
Ext: 360
Email: glahai@mrc.gm
Dr Yin Bun Cheung
E mail YinBun.Cheung@lshtm.ac.uk

Data entry: Yankuba Barrow, Yamusu Nyang

Signature page

Sponsor's representative

Prof Tumani Corrah<br>MD, PhD, FRCP, PWACP	Date:	Signature:

Principal investigator

Prof Richard Adegbola<br>PhD, FRCPath	Date: 17 August 2006	Signature: 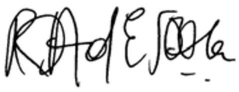

Safety Monitor

Dr. Ousman Nyang

Abbreviations

**AE**	Adverse Event/Adverse Experience
**AR**	Adverse Reaction
**ARI**	Acute Respiratory infection
**CRF**	Case Report Form
**EPI**	Expanded Programme of immunisation
**GCP**	Good Clinical Practice
**IB**	Investigator's Brochure
**ICF**	Informed Consent Form
**ICH**	International Conference on Harmonization
**ICMJE**	International Committee of Medical Journal Editors
**ID**	Identification
**IEC**	Independent or Institutional Ethics Committee
**IRB**	Institutional Review Board
**MRC**	Medical Research Council
**N**	Number (typically refers to subjects)
**NPS**	Nasopharyngeal Swab
**NVT**	Non Vaccine Type
**PCV**	Pneumococcal Conjugate Vaccine
**PI**	Principal Investigator
**SAE**	Serious Adverse Event/Serious Adverse Experience
**SAR**	Serious Adverse Reaction
**SCC**	Scientific Coordinating Committee
**SmPC**	Summary of product Characteristics
**SOP**	Standard Operating Procedure
**TSC**	Trial Steering Committee

Table of Contents

Study Management **6**

Schema **5**

1.0 INTRODUCTION 8

2.0 STUDY OBJECTIVES 8

3.0 SELECTION AND ENROLLMENT OF SUBJECTS 8

4.0 CLINICAL AND LABORATORY EVALUATIONS 11

5.0 DATA COLLECTION & MONITORING AND ADVERSE EVENT REPORTING 16

6.0 STATISTICAL CONSIDERATIONS 17

7.0 HUMAN SUBJECTS 18

8.0 PLANS FOR DISTRIBUTION OF FINDINGS TO STUDY COMMUNITY 18

9.0 BIOHAZARD CONTAINMENT 19

APPENDICES 20

Study Management

All questions concerning this protocol should be sent via e-mail to uegere@mrc.gm.

For Questions About Study Subjects, The Schedule Of Events, Case Report Forms, Entering Waivers For Randomisation/Registration Exemptions, Transfers, And Delinquencies, And Other Data Management Issues, the Project Data Manager will respond:

Send an e-mail message to uegere@mrc.gm or phill@mrc.gm
Include the subject ID
Give a detailed description of the question

For Adverse Events Questions:

Send an e-mail message to uegere@mrc.gm
Call MRC ext 441

Schema

Title:	Community Randomised Controlled Trial to Assess the Impact of Vaccination with a Pneumococcal Conjugate Vaccine on Nasopharyngeal Carriage of Pneumococci in The Gambia
Short title	Sibanor Vaccine Trial
Phase:	IV
Population:	Approximately 6000 residents of 21 Gambian villages for vaccination<br>Subsamples of 1250 subjects for evaluation antigenic and molecular characteristics of pneumococci with a subsample of 200 for 5ml blood specimen and saliva.<br>All children born during the study period<br>Subsample of 430 subjects for longitudinal substudy
Number of Sites:	Sibanor villages<br>MRC Labs, Fajara, The Gambia
Study Duration:	36 months (June 2006 until May 2009)
Subject Participation Duration:	Up to 24 months
Description of Products or Intervention:	Seven-valent pneumococcal conjugate vaccine (Prevenar) and meningococcal polysaccharide C conjugate vaccine
Objectives:	1. To determine the impact of immunisation of a whole community with Prevenar on nasopharyngeal carriage of pneumococci.<br>2. To determine the effects of vaccination on the prevalence, density, duration and rate of acquisition of nasopharyngeal colonisation with pneumococci of vaccine serotype and of non-vaccine serotype.<br>3. To determine changes in the antigenic and molecular characteristics of pneumococci obtained from the nasopharynx of vaccinated and control subjects.
Description of Study Design:	This schematic should provide an overview of the study design, including study arms, sample size and schedule of interventions (e.g. vaccine administration), if applicable; a detailed schematic describing all visits and assessments (schedule of events) will be included in Appendix A.
Estimated Time to Complete Enrolment:	3 months

1.0 INTRODUCTION

1.1 Background

The prevention of pneumococcal disease in young children is a major, international public health priority. Acute lower respiratory tract infections (ALRI) are responsible for about 4 million deaths a year in children; the majority of these deaths occur in the developing world[1]. *Streptococcus pneumoniae* is the most important cause of severe ALRI in children and the likely cause of many of these deaths[2]. In addition *S. pneumoniae* is a major cause of meningitis and otitis media[3]. Susceptibility to pneumococcal infection is increased by HIV infection and the incidence of pneumococcal infection in children is increasing in sub-Saharan Africa[4]. Asymptomatic nasopharyngeal carriage of pneumococci is common in many developing countries, where colonisation of the nasopharynx with pneumococci commences shortly after birth[5] and, by the age of two years, almost all children are carriers[6,7]. Carriage rates remain relatively high in older children and in adults[7], who may be an important source of pneumococcal infection in young children.

Studies undertaken in a number of developing countries have shown that mortality from pneumonia can be reduced significantly by active case detection and treatment of children with an ALRI with an antibiotic[8]. However, such programmes are difficult to sustain and they are threatened by the rapid spread of pneumococci that are resistant to widely used antibiotics such as co-trimoxaxole and penicillin[9]. The success of an *Haemophilus influenzae* type b (Hib) polysaccharide/protein conjugate vaccine in reducing the overall incidence of radiological pneumonia in children in The Gambia and in Chile[10,11] suggests that an effective pneumococcal vaccine would be even more effective.

Pneumococcal polysaccharide vaccines are effective in preventing invasive pneumococcal disease in immunocompetent adults[12] but their ability to prevent pneumonia is uncertain. [13] They are poorly immunogenic in young children[14] and have little effect on the incidence of otitis media or nasopharyngeal carriage[15,16]. Thus, it was surprising that a 23-valent pneumococcal polysaccharide vaccine reduced mortality from pneumonia in children in Papua New Guinea, even in children under the age of two years[17]. However, this study has not been repeated and attention is now being directed to polysaccharide/protein conjugate vaccines. Pneumococcal conjugate vaccines are immunogenic in infants and induce immunological memory. Following a series of pilot studies, which demonstrated the safety and immunogenicity of pneumococcal conjugate vaccines when given during the first six months of life[18], a number of large-scale efficacy trials have been undertaken. In California, a seven-valent pneumococcal conjugate vaccine given to infants at the ages of 2, 3 and 4 months[19] reduced the incidence of invasive pneumococcal disease caused by pneumococci of vaccine serotype by over 90%, the incidence of radiological pneumonia by 33% and the overall incidence of otitis media by 7%. A large, cluster-randomised trial of a seven-valent pneumococcal conjugate vaccine in Navajo and White Mountain Apache children showed a 77% reduction in the incidence of invasive pneumococcal disease in vaccinated children but no significant effect on the incidence of pneumonia. [20] In contrast, in South Africa significant protection against both invasive pneumococcal disease (vaccine efficacy 83% [95% CI 39.97]) and pneumonia (vaccine efficacy 20% [95% CI 2,35]) was observed among HIV negative children immunised with a nine-valent vaccine. Protection was less in HIV positive children. [21] In Finland, a seven-valent pneumococcal conjugate vaccine reduced the incidence of serotype specific otitis media by 57%[22]. In The Gambia a large trial involving17,400 infants given three doses of a nine-valent conjugate or control vaccine at the ages of 2, 3 and 4 months has recently been completed. The pneumococcal conjugate vaccine gave protective efficacies of 37% against radiological pneumonia and 77% against invasive pneumococcal disease caused by vaccine serotypes. In addition, the vaccine reduced morbidity and mortality among vaccinated children by 15% and 16% respectively despite

preventing only 50% of culture- proven pneumococcal disease[23]. The results of the Gambian trial highlight both the potential value of using existing conjugate vaccines in The Gambia and elsewhere and the need for additional research on pneumococcal disease prevention. A further trial is being conducted in the Philippines with an eleven-valent vaccine and will be completed at the end of this year.

On the basis of the results obtained in California, the seven-valent pneumococcal conjugate vaccine Prevenar [R] produced by Wyeth Lederle was introduced into routine use in the United States in 2000. The results have been dramatic; not only has invasive pneumococcal disease almost disappeared in the vaccinated paediatric population but the incidence of invasive pneumococcal disease has fallen markedly in the elderly and in young adults, [24] presumably because of a reduction in the spread of pneumococci of vaccine type (VT) from vaccinated infants. This hypothesis is supported by the results of nasopharyngeal carriage studies, which have shown a reduction in the prevalence of nasopharyngeal carriage with pneumococci of VT in contacts of vaccinated infants. [25]

A reduction in the prevalence of nasopharyngeal carriage of pneumococci of VT was found in Gambian infants immunised with a five-valent pneumococcal conjugate vaccine during the first few months of life and then re-immunised with a 23-valent pneumococcal polysaccharide vaccine at the age of two years[26]. However, this was accompanied by an increase in the carriage rate of pneumococci of non-vaccine type (NVT), the first time that this phenomenon was shown, and the overall prevalence of pneumococcal carriage in vaccinated children was little changed. Children in this study had very high antibody concentrations at the time that nasopharyngeal swabs were collected because of their booster immunisation. A study of Gambian children one and four months after completion of a primary course of vaccination with a nine-valent conjugate vaccine at the ages of 2, 3 and 4 months showed a similar trend towards the replacement of pneumococci of vaccine serotype with those of non-vaccine serotype[27] but the effect was not as marked as in the initial study, perhaps because these children had lower antibody concentrations. An initial study in Israel did not show serotype replacement[28] but subsequent studies done in South Africa and in Israel have done so [29,30]In the second Israeli study, which was undertaken in children attending day care centres, pneumococci of NVT were found infrequently in very young children and remained uncommon in non-vaccinated children. However, in vaccinated children the proportion of NVT pneumococci relative to those of VT isolated from the nasopharynx increased gradually with age and, by the age of three years, NVT pneumococci predominated[30]. In American Indians, while the reduction in the carriage of VT pneumococci was similar across all ages after vaccination, the carriage of NVT pneumococci was more marked in adults, accounting for an increased in the overall carriage rate[20]. Subsequently, several further studies have shown replacement of VT pneumococci by those of non-vaccine serotypes in the nasopharynx of vaccinated children.[31] Because high levels of antibiotic resistance are found most frequently among pneumococci of vaccine serotype, vaccination with pneumococcal conjugate vaccines has led to a reduction in the prevalence of antibiotic resistant pneumocococi in vaccinated children [31] and, in the United States, introduction of infant immunisation with a pneumococcal conjugate vaccine has had an impact on the overall prevalence of antibiotic resistant pneumococci.[32] Thus, there are clear indications that serotype replacement as a consequence of vaccination can have indirect beneficial effects. Could serotype replacement in the nasopharynx also have undesirable consequences?

The non-vaccine serotypes of pneumococci found most frequently in vaccinated children, for example serotypes 3, 7F, 8, 12F, 45 and 46, are capable of causing invasive disease in experimental animals and, in the absence of vaccination, occasionally caused invasive disease in man. For this reason concerns were expressed shortly after the replacement effect was first observed that there was a risk that widespread introduction of a vaccine directed against only a limited number of serotypes might result in an increase in the incidence of invasive disease caused by pneumococci of NVT. [33] Initial experience in California was reassuring but, in 2001, Finnish scientists reported an increase of 37% in the incidence of otitis media caused by pneumococci of NVT in children who had received a pneumococcal conjugate vaccine. [22]

However, until recently there was no evidence for an increase in the incidence of invasive pneumococcal disease caused by pneumococci of NVT in vaccinated populations. Overall experience in the United States since the introduction of Prevenar [R] has been reassuring but a small increase in the incidence of invasive disease caused by pneumococci of NVT is now being seen in the paediatric population. Numbers of cases are still very small and the overall benefit of vaccination is still overwhelming. [34] However, the recent finding of a marked and statistically significant increase in the incidence of invasive pneumococcal disease due to pneumococci of NVT among HIV positive subjects in the USA, [35] the effect being especially marked in females, is a matter of considerable concern, especially for developing countries where the prevalence of HIV infection is high and pneumococcal disease rampant.

The mechanism of serotype replacement in the nasopharynx is not fully understood. It is possible that initially the immune response induced by vaccination provides a selective advantage to pneumococci of NVT present in a mixed population of pneumococci in which they had previously been suppressed. Subsequently, high antibody levels may prevent colonisation by VT pneumococci. In the presence of high antibody concentrations against selected capsular polysaccharides, pneumoccal clones, which have other advantageous survival characteristics, may undergo capsular switching to a non-vaccine serotype. [36]

The pneumococcal conjugate vaccine trial recently completed in The Gambia employed a nine-valent conjugate vaccine which includes serotypes 1 and 5 conjugates that are not present in Prevenar [R]. Recent studies from Israel have shown that invasive infections caused by pneumococci belonging to these serotypes are especially severe. [37] Unfortunately, development of the nine-valent vaccine used in The Gambian trial has been stopped and, although at least one other multiple valent vaccine is being developed, the only vaccine likely to be available for routine use for a number of years is seven-valent Prevenar [R]. In the USA where a large fall in vaccine-type invasive disease has been observed following the introduction of Prevenar [R] small increases in non-vaccine type invasive disease are being recorded even among unvaccinated populations but overall the effect on health is still positive.

1.2 Rationale

In The Gambia and other developing countries where children acquire pneumococci at a very early age, where the prevalence of colonization and the incidence of invasive disease are high and where the dynamics of transmission differ from the United States, it is unclear what impact the widespread use of pneumococcal conjugate vaccines will have on transmission between vaccinated and unvaccinated populations. Therefore, a study of the epidemiology of pneumococcal carriage has started in 21 rural Gambian villages to examine the dynamics of pneumococcal carriage and transmission. The main objective of this study is the evaluation of the effects of pneumococcal vaccination on carriage of pneumococci, particularly serotypes 1 and 5, using a community randomised approach. Although pneumococci of serotypes 1 or 5 are important causes of invasive disease in The Gambia, and many other developing countries, they are found infrequently in the nasopharynx. Could introduction of a seven-valent vaccine enhance their ability to establish themselves in the nasopharynx and subsequently to cause invasive disease? Although the introduction of a seven valent vaccine into communities where type 1 and type 5 pneumococcal disease is common will almost certainly result in a reduction in the overall incidence of invasive pneumococccal disease an increase in the incidence of invasive disease caused by these virulent pneumococci could detract from its overall public health benefit.

For these reasons we believe that it is essential to determine whether the introduction of Prevenar [R] into a community where type 1 and type 5 pneumococci are important causes of invasive disease will result in an increase in the nasopharyngeal carriage rate of these and other NVT pneumococci. In order to study the effects of maximum immune pressure of the kind that will be seen only several years after the introduction of a pneumoocccal conjugate

vaccine into the infant immunisation programme we propose to study the effects of vaccination of a whole community as well as that of vaccinating just the infant population.

1.3 Potential Risks and Benefits

Prevnar has not been formally licensed for use in adults, although other studies have given this vaccine to adults. Secondly it is possible that serotype replacement will occur and this could ultimately lead to increased carriage of some pathogenic serotypes in the nasopharynx.

It is likely that some individuals who might otherwise have developed invasive pneumococcal disease will not do so as a benefit of receiving this vaccine. Furthermore, because of the involvement of MRC in the villages, there may well be a health benefit for the participants involved in the study.

1. Leowski J. Mortality from acute respiratory infections in children under five years of age: global estimates. Wld Hlth Statist Quart 1986;39: 138-44.
2. Greenwood BM. The epidemiology of pneumococcal infection in children in the developing world. Phil Trans R Soc Lond B 1999;354:777-85.
3. Berman S. Otitis media in children. N Engl J Med 1995;332:1560-5.
4. Jones N, Huebner R, Khoosal M, Crewe-Brown, Klugman K. The impact of HIV on *Streptococcus pneumoniae* bacteraemia in a South African population. AIDS 1998;12; 2177-84.
5. Gratten M, Gratten H, Poli A, et al. Colonisation of *Haemophilus influenzae* and *Streptococcus pneumoniae* in the upper respiratory tract of neonates in Papua New Guinea; primary acquisition, duration of carriage, and relationship to carriage in mothers. Biol Neonate 1986;50:114-20.
6. Frederiksen B, Henrichsen J. Thorat carriage of *Streptococcus pneumoniae* and *Streptococcus pyogenes* among infants and children in Zambia. J Trop Pediatr 1988;34:114-117.
7. Lloyd-Evans N, O'Dempsey TDJ, Baldeh I, et al. Nasopharyngeal carriage of pneumococci in Gambian children and in their families. Peditr Infect Dis J 1996;15:866-71.
8. Sazawal S, Black RE. Meta-analysis of intervention trials of case-management of pneumonia. Lancet 1992;340:528-33.
9. Thornsberry C, Sahm DF. Antimicrobial resistance in respiratory tract pathogens: results of an international surveillance study. Chemotherapy 2000;46 Suppl 1:15-23.
10. Mulholland K, Hilton S, Adegbola R, et al. Randomised trial of *Haemophilus influenzae* type-b tetanus protein conjugate for prevention of pneumonia and meningitis in Gambian infants. Lancet 197;349:1191-97.
11. Lagos R, Horwitz I, Toro J, et al. Large scale, postlicensure, selective vaccination of Chilean infants with PRP-T conjugate vaccine: practicability and effectiveness in preventing invasive *Haemophilus influenzae* type b infections. Pediatr Infect Dis J 1996;15:216-22.
12. Fedson DS. The clinical effectiveness of pneumococcal vaccination: a brief review. Vaccine 1999;17: S85-90.
13. Mangtani P, Cutts F, Hall AJ. Efficacy of polysaccharide pneumococcal vaccine in adults in more developed countries; the state of the evidence. Lancet Infect Dis 2003;3:71-8.
14. Temple K, Greenwood BM, Inskip H, et al. Antibody response to pneumococcal polysaccharide vaccine in African children. Pediatr Infect Dis J 1991;10:386-90
15. Douglas RM, Miles HB. Vaccination against *Streptococcus pneumoniae* in childhood: lack of demonstrable benefit in young Australian children. J Infect Dis 1984:149: 861-9.
16. Douglas RM, Hansman D, Miles HB, Paton JC. Pneumococcal carriage and type-specific antibody. Failure of a 14-valent vaccine to reduce carriage in healthy children. Amer J Dis Child 1986;140:1183-5.
17. Riley ID, Lehman D, Alpers MP, et al. Pneumococcal vaccine prevents death from acute-lower-respiratory-tract infections in Papua New Guinea. Lancet 2986;ii:877-81.
18. Eskola J, Antilla M. Pneumococcal conjugate vaccines. Pediatr Infect Dis J. 1999;18:543-51.
19. Black S, Shinefield H, Ray P, et al. Efficacy of heptavalent conjugate pneumococcal vaccine (Wyeth Lederle) in 37,000 infants and children: impact on pneumonia, otitis media and an up-date on invasive disease – results extended follow-up of the efficacy trial cohort. Second International Symposium on Pneumococci and Pneumococcal Disease, Sun City, South Africa, March 19-23, 2000, abstract 019.
20. O'Brien KL, Moulton LH, Reid R, et al. Efficacy and safety of seven-valent conjugate pneumococcal vaccine in American Indian children: group randomised trial. Lancet 2003; 362:355-61.
21. Klugman KP, Madhi SA, Huebner RE, et al. A trial of a 9-valent pneumococcal conjugate vaccine in children with and those without HIV infection. NEJM 2003; 349: 1341-8.
22. Kipli T, Jokinem J, Herva E, et al. Effect of hepatavalent pneumococcal conjugate vaccine (PNCCRM) on pneumococcal otitis media (AOM) by serotype. Second International Symposium on Pneumococci and Pneumococcal Disease, Sun City, South Africa, March 19-23 2000, abstract O20.
23. Cutts,F T, Zaman SM, Enwere G, et al.. Efficacy of nine-valent pneumococcal conjugate vaccine against pneumonia and invasive pneumococcal disease in The Gambia: randomised, double-blind, placebo-controlled trial. Lancet 2005;**365**(9465): 1139-46.

24. Whitney CG, Farley MM, Hadler J. et al. Decline in invasive pneumococcal disease after the introduction of protein-polysaccharide conjugate vaccine. N Engl J Med 2003;348:1737-46.
25. Hammitt LL, Bruden D, Butler JC et al. Indirect effect of childhood heptavalent pneumococcal conjugate vaccination on adult carriage of *Streptococcus pneumoniae*: a community study. Presentation ISPPD-4 2004, May 9-13, Helsinki
26. Obaro SK, Adegbola RA, Banya WAS, Greenwood BM. Carriage of pneumococci after pneumococcal vaccination. Lancet 1996;348:271- 2.
27. Obaro SK, Adegbola RA, Chang IH, et al. Safety and immunogenicity of a nano-valent pneumococcal vaccine conjugated to CRM_{197} administered simultaneously but in a separate syringe with diphtheria, tetanus and pertussis vaccine in Gambian infants. Pediatr Infect Dis J 2000;19:463-9.
28. Dagan R, Melamed R, Muallen M, et al. Reduction of nasopharyngeal carriage of pneumococci during the second year of life by a hepatavalent pneumococcal conjugate vaccine. J Infect Dis 1996;174:1271-83.
29. Mbellee N, Huebner R, Wasas AD, et al. Immunogenicity and impact on nasopharyngeal carriage of a nonavalent pneumococcal conjugate vaccine. J Infect Dis 1999;180:1171-6.
30. Dagan R. Effect of vaccine on antibiotic- resistant *S. pneumoniae* (PNC) carriage and spread. Second International Symposium on Pneumococci and Pneumococcal Disease, Sun City, South Africa, March 19-23 2000, abstract 072.
31. Klugman KP Efficacy of pneumococcal conjugate vaccines and their effect on carriage and antimicrobial resistance. Lancet Infect Dis 2001;1:85-91.
32. Stephens DS, Zughaier S, Whitney CG et al. Rapid decline in macrolide resistance in *Streptococcus pneumoniae* following introduction of the pneumococcal conjugate vaccine: a population-based assessment. Presentation ISPPD-4 2004, May 9-13, Helsinki
33. Spratt BG, Greenwood BM. Prevention of pneumococcal disease by vaccination: does serotype replacement matter ? Lancet 2000;356:1210-1.
34. Whitney C. Effect of pneumococcal conjugate vaccine onm invasive disease in the U.S. . Presentation ISPPD-4 2004, May 9-13, Helsinki.
35 Schuchat A, Flannery B, Heffernan R et al. Invasive pneumococcal disease in HIV –AIDS: has introduction of 7-valent pneumococcal conjugate vaccine (PCV-7) reduced HIV-related disease burden in the USA ? . Presentation ISPPD-4 2004, May 9-13, Helsinki.
36. Coffey TJ, Enright MC, Daniels M, et al. Recombinant exchanges at the capsular polysaccharide biosynthetic locus lead to frequent serotype changes among natural isolates of *Streptococcus pneumoniae*. Mol Microbiol 1998;27:73-83.
37 Dagan R, Givon-Lavi N, Bar-Ziv J. et al. The association of nasopharyngeal carriage (NP) of *S pneumoniae* with community-acquired alveolar pneumonia (CAAP) determined by WHO standardization of interpretation of chest radiographs in children (WHO-SICR). Poster presentation ISPPD-4 2004, May 9-13, Helsinki.
38. Lafong AC, Crothers E. Simple latex agglutination method for typing pneumococci. J Clin Path 1988;41:230-1.
39. Jacobs MR, Bajakssouzian S, Appelbaum PC, Bolstrum A. Evaluation of the E test for susceptibility testing for pneumococci. Diagn Microbiol Infect Dis 1992;15:473-8.
40. Roszak DB, Grimes DJ, Colwell RR. Viable but nonrecoverable stage of *Salmonella enteritidis* in aquatic systems. Can J Microbiol 1984;30:334-8.
41. Enright M, Spratt BG. A multilocus sequence typing scheme for *Streptococcus pneumoniae*: identification of clones associated with serious invasive disease. Microbiology 1998;144:3049-60.
42. Zhou J, Enright MC, Spratt BG. Identification of the major Spanish clones of penicillin-resistant pneumococci via the Internet using multilocus sequence typing. J Clin Microbiol 2000;38:977-86.

1.4 Study Design

This study is a single-blind placebo-controlled community randomized trial. All residents of selected 21 Gambian villages are eligible including those born during the study.

All children of the trial community between 2 months of age up to the age of 30 months will receive the seven-valent pneumococcal conjugate vaccine (PCV):

- Subjects born during the study will receive three doses of the vaccine to be given monthly at the ages of 2, 3 and 4 months.
- Subjects between the age of 2 and 11 months will receive three doses of the vaccine to be given monthly
- Children aged between 12 and 30 months will receive two doses at monthly intervals.

The 21 villages will be randomly assigned to one group of 10 villages and one group of 11 villages according the randomisation SOP. All subjects above the age of 30 months will receive either one dose of PCV or one dose of a meningococcal polysaccharide C conjugate vaccine. The vaccination campaign will be village by village according to an SOP.Because of vaccine pre-labelling differences, nurse field workers administrating vaccine will be able to tell which vaccine they are giving. The field team will not communicate the vaccine allocation to other parties. Biological samples will have unique identifiers and will show no information regarding the vaccine received or the participants. Safety of vaccination will be monitored by nurse fieldworkers and study clinician according to an SOP.

3, 12 and 24 months after the first vaccination, cross-sectional surveys will be conducted. The sample for each survey will be obtained through a random sampling method that ensures coverage of all age groups and all villages. This will be described in detail in an SOP. Each subject will have an NPS taken. 5ml blood and saliva specimens will be obtained from 200 subjects, selected according to a corresponding SOP before vaccination and then at the 3, 12 and 24 month time-points. The selected subjects will be distributed across the study villages stratified by age to ensure that 50 come from children in the age range 30 months to 5 years, 50 from children 6 to 15 years, 100 from those aged >15 years.

NPS will be collected from all children born in the population during the study period. An NPS will be collected as soon as possible after birth and then at weekly intervals until the first dose of vaccine at 2 months.

One hundred and twenty children 31-59 months of age, 130 6 to 15 years old and 180 adults >15 years, will be selected randomly (according to an SOP). NPS will be obtained monthly from these subjects for 6 months and then 3 monthly until the end of the 24 months followup.

2.0 STUDY OBJECTIVES

The study objectives are:

- To determine the impact of immunisation of a whole community with a pneumococcal polysaccharide/protein conjugate vaccine on nasopharyngeal carriage of pneumococci.

- To determine the effects of vaccination on the prevalence, density, duration and rate of acquisition of nasopharyngeal colonisation with pneumococci of vaccine serotype and of non-vaccine serotype.

- To determine changes in the antigenic and molecular characteristics of pneumococci obtained from the nasopharynx of vaccinated and control subjects.

3.0 SELECTION AND ENROLLMENT OF SUBJECTS

3.1 Inclusion Criteria

- Resident of one of twenty one Gambian villages

3.2 Exclusion criteria

- Failure of the family/subject to give consent
- non-residence in the villages,
- declared intent of the family/subject to leave the study area permanently within the following 3 months
- Previous exposure to a conjugate pneumococcal vaccine

A record will be kept of subjects who are excluded and the reason for their exclusion will be noted on a form.

3.3 Enrollment Procedures

Subjects will be recruited from twenty-one Gambian villages. The purpose of the study will be described at meetings with village elders and other members of the village and what it involves for participants. Before enrolling individual members of the villages the nature of the trial will be fully explained including the treatment, observation details, spectrum of likely side effects, follow-up details and extent of collection of Nasopharyngeal swabs (NPS), saliva and blood sampling.

3.4 Substudies

Sub studies will take place; these will be all covered in revised consent forms, once approved.

3.5 Co-enrollment Guidelines:

As is MRC policy, study subjects are encouraged not to be involved in other studies.

4.0 CLINICAL AND LABORATORY EVALUATIONS

4.1 Pre-entry/Entry Evaluations

4.11 Screening

Details of the study will be carefully discussed with the subjects, who will be asked to read, or will be read to, and sign (or thumb-print) an informed consent form prior to any study-related evaluations being performed. Translations into the local languages will be provided. The subjects that agree to enroll and have signed consent documentation will be enrolled for the study.

4.12 Entry

Subjects will be excluded based on the criteria enumerated above (Section 3.2).

4.2 Evaluations During study

4.2.1 Screening at the villages

- Inclusion/exclusion criteria will be checked.
- Written informed consent will be obtained.
- Subject Exclusion Form will be filled by the field supervisor for all subjects excluded from the study
- A nurse field worker will fill the Subject Status Form for all subjects recruited
- Nurse field workers will administer conjugate pneumococcal vaccine to all children up to the age of 30 months.
- In group 1 villages, subjects older than 30 months will receive one dose of conjugate pneumococcal and their equivalent in group 2 villages will receive one dose of the meningococcal polysaccharide C conjugate vaccine.
- Vaccine ID will be included on subjects identity card
- All subjects will be asked to report all health related complaints to the field team.
- 5ml blood and saliva specimen will be obtained from 200 selected subjects following the corresponding SOP.
- Specimens will be put in cool boxes and transferred to the MRC microbiology laboratory on the same day as collection.
- For any specimen, if an individual refuses, we will select the next person on the list supplied by the data room. For those who are travelled we will try and capture them for sampling but after 2 weeks if unsuccessful, will sample the next person on the list.

4.21 Scheduled follow-up

Infants aged between 2 and 11 months will receive two additional doses of the pneumococcal conjugate vaccine (PCV) at monthly intervals.
Children aged between 12 and 30 months will receive a second dose of PCV a month after the initial dose.

At three, twelve and twenty-four months after the scheduled round of vaccination, nasopharyngeal swabs will be collected from approximately 1,250 subjects, ensuring coverage of each age group and all villages in the cross-sectional survey. These subjects will be selected randomly as described in section 1.4 and separate randomisation procedures will be undertaken for each survey; subjects included in a previous survey will not be excluded. 5ml blood and saliva specimens will be obtained from 200 selected subjects as described in section 1.4.

NPS will be collected weekly from all new births into all the villages until the age of 2 months when they receive the first doses of the pneumococcal conjugate vaccine

NPS will be collected monthly from 430 selected subjects as described in section 1.4.

All new births into the twenty-one villages will receive three doses of pneumococcal conjugate vaccine at monthly intervals from the age of two months.

In the lifetime of the study, new settlers in the villages will be identified and recruited for the study. The same vaccination schedule will apply.

All laboratory procedures will be described in SOPs.

4.3 Subjects who prematurely discontinue the study

Subjects, who leave the study at any time, may do so without repercussions. They may still access medical care provided by the study team, and will not be turned away.

5.0 DATA COLLECTION AND MONITORING AND ADVERSE EXPERIENCE REPORTING

5.1 Records to Be Kept

A regulatory folder and a protocol deviations folder will be established.

All filled in paper forms will be filed in securely by the data manager. Individual medical records will be filed together. All electronically stored data will be stored securely and backed up by the data manager.

5.2 Role of Data Management

The Data manager will be responsible for receiving, entering, cleaning, querying, analysing, and storing the data, which accrues from the study. He will generate queries to be addressed by the investigators and will be responsible for linking the paper and electronic data from the field and the clinic with the paper and electronic laboratory data from the immunology, microbiology, haematology and molecular biology laboratories.

5.4 Adverse events reporting

Everyone vaccinated will be requested to report untoward events to the study team. Adverse events will be recorded as observed by the Investigators or as volunteered by the subject or his/her parent/guardian. Full details will be documented in the CRF whether or not the Investigator or his deputies consider the event to be related to the trial substance.

Serious adverse events (SAEs) that occur during the study or within six months of the vaccinations will be notified immediately by telephone by the principal investigator to the study Safety Monitor. All SAEs identified will then be reported jointly by the research clinician and P. Hill to the Principal investigator and the study Safety Mmonitor as well as to Wyeth, the vaccine manufacturer, accordingly.

Serious adverse events are defined as an event that:

- results in death;
- is life-threatening (*i.e.,* the subject was at risk of death at the time of the event);
- requires or prolongs in-patient hospitalisation;
- results in persistent or significant disability/incapacity;
- is a congenital anomaly/birth defect.

Minimum details to be given in a telephone report are:

- Name of reporting doctor and contact telephone number.
- Study number.
- Nature of adverse event.
- Subject details (number, initials, sex, date of birth, weight and age).
- Date and time of event.
- Drug history.
- Other relevant history.
- Outcome.
- Causality.

The event will be documented on the SAE page of the CRF and reported as appropriate. After the ethics committee's response to the SAE is received, the Principal Investigator and Clinical Monitor will meet to determine the future plan for the study, which could involve amending the protocol, discontinuing the administration of the vaccine, or continuing unchanged for the other volunteers.

6.0 STATISTICAL CONSIDERATIONS

6.1 General Design Issues

Villages will be randomly assigned to a study group. A computer-generated list of random numbers will be used to assign villages to PCV group (Group 1) or Mening C group (Group 2). Nurse field workers will administer the vaccines.

6.2 Outcomes of interest

6.21 Impact on carriage

(a) Differences in the prevalence of nasopharyngeal carriage of pneumococci of vaccine or of non-vaccine serotype in study villages after vaccination in the cross-sectional samples. Differences in the prevalence of carriage with serotype 1 and serotype 5 pneumococci will be of particular importance.

(b) Acquisition rates of nasopharyngeal carriage in newborns resident in vaccinated or control villages.

(c) Rate of capsular switching in villages where community wide pneumococcal conjugate vaccination has been introduced, compared to those receiving placebo.

6.22 Pneumococcal IgG and IgA in serum and saliva

This will be determined yes/no antibody assay result, and compared to the carriage results.

6.3 Sample Size and Accrual

The baseline survey showed that the prevalence of nasopharyngeal carriage with pneumococci of the seven valent vaccine serotype was about 40% in infants up to the age of 2.5 months (the age when the first dose of pneumococcal conjugate vaccine will be given) and 30% in those aged 30 months or more. The coefficient of variation between villages was 0.3. Based on these assumptions and assuming that 50 subjects are studied in each village the trial will have over 90% power with a type 1 error of 5% to detect a 50% reduction in the overall prevalence of pneumococci of vaccine serotype in those over the age of 30 months (prevalence 30% to 15%) and a similar power to detect a 50% reduction in the colonisation of infants with pneumococci of vaccine type before the age of 2 months (prevalence reduced from 40% to 20%. The prevalence of type 1 and type 5 carriage was very low in the initial survey so the study is not specifically powered to detect an increase in carriage with pneumococci of these serotypes but it would detect this if the effect was marked.

6.4 Monitoring and Analysis

The MRC Clinical Trials monitor will be responsible for monitoring the trial. This will include site visits to confirming the existence of the appropriate documents in the regulatory folder and of source documents for all required data. She will also assess the accuracy and

completeness of the CRF entries and other records against each other and their consistency with the source documents.

The data manager will be responsible for data entry, data cleaning and for initial analysis of the results. The main analyses will be descriptive and comparative.

7.0 HUMAN SUBJECTS

7.1 Institutional Review Board (IRB) Review and Informed Consent

Approval for the study has been given by the joint Gambia government/MRC ethics committee. Written informed consent will be obtained from the subject (or parent, legal guardian, or person with power of attorney for subjects who cannot consent for themselves, such as, those below the legal age). The subject's assent will also be obtained if he or she is able to understand the nature, significance and risks associated with the study. The informed consent will describe the purpose of the study, the procedures to be followed, and the risks and benefits of participation. A copy of the consent form will be given to the subject (or parent or legal guardian).

7.2 Subject Confidentiality

All records will be kept in a secure place. All data on computer files will have restricted access. Clinical information will not be released without written permission of the subject, except as necessary for monitoring.

7.3 Data and Safety Management Board (DSMB)

A data and safety management board has been constituted for the trial and a schedule for meetings will be agreed to. DSMB includes the following people: Dr Anthony Scott (Kilifi, Kenya), Dr Ayo Palmer (Banjul), Dr Derrick Crook (Oxford University) and Dr Dongmei Liu (London School of Hygiene and Tropical Medicine). A clinical safety monitor will be selected and a programme of trial monitoring will be set up for the trial.

7.4 Study Discontinuation

The MRC SCC or the joint Gambian government/MRC ethics committee may discontinue the study after the appropriate recommendation by DSMB.

8.0 PLANS FOR DISTRIBUTION OF RESEARCH FINDINGS TO STUDY COMMUNITY

Written annual progress reports of the trial will be produced. At the end of the study, a research report of the methods, detailed results, and brief conclusions will be prepared for distribution to the collaborators. A simplified lay document will be made available to study subjects.

9.0 BIOHAZARD CONTAINMENT

As the transmission of HIV and other blood-borne pathogens can occur through contact with contaminated needles, blood, and blood products, appropriate blood and secretion precautions will be employed by all personnel in the drawing of blood and shipping and handling of all specimens for this study, according to the MRC safety manual.

Appendices

GAMBIAN COMMUNITY PNEUMOCOCCAL CONJUGATE VACCINE TRIAL-
CONSENT PROCEDURE

1. The project investigators will hold discussions with village heads and elders in villages in the study area to determine if they are interested in participating in the study.

2. If village elders are encouraging, village meetings will be held, which will be open to the whole community. At these meetings the nature of the trial and what it involves for the village will be discussed. General consent for a village to join the trial will be sought at the meeting and if this is given, this will be documented formally.

3. In villages that agree to join the trial, the head of each compound will be visited. The nature of the trial and what this will involve for his household will be explained by a trained field assistant and an opportunity will be given to ask questions. If the compound head wants his household to join the trial, information sheets will be provided and explained after which he/she will be asked to sign a consent form (Consent form 1).

4. At the time of vaccination, study individuals or the guardians of small children will be asked for their consent to vaccination. The fact that the respondent gave permission will be recorded on a special form by the field assistant who asks for permission

5. Whenever a nasopharyngeal swab or blood sample is collected, the study subject or guardian will be reminded that they are under no obligation to provide the sample and that they can withdraw from the study at any time. If permission is given this will be noted by the field assistant on a form.

6. Subjects asked to join the longitudinal study in which nasopoharyngeal swabs will be collected will be asked to sign a separate consent form (Consent form 2)

MEDICAL RESEARCH COUNCIL (MRC)/GAMBIAN COMMUNITY PNEUMOCOCCAL CONJUGATE VACCINE TRIAL

INFORMATION SHEET TO BE EXPLAINED TO PARENTS AND ADULT PARTICIPANTS

Pneumonia, an infection of the lungs and meningitis an infection of the brain, are both serious diseases in The Gambia causing many deaths and much serious illness. These conditions are most frequent in young children but can also affect adults.

The germs that cause pneumonia and meningitis are often found in the throat of people who are quite healthy but the germs can spread from these healthy people to other people who may then become seriously sick. The germs have many different types. MRC is working with the Government of The Gambia on the evaluation of vaccines to prevent some kinds of pneumonia and meningitis. A successful trial of a pneumonia vaccine was concluded in URD and CRD last year.

MRC would want to know whether or not there would be changes in the types of this germ in the throat when the vaccine is used in a large scale in the future. You and members of your family are invited to take part in the study.

All children up to the age of 30 months will receive 3 doses, at one monthly interval, of a pneumococcal vaccine that has been licensed and in use in the USA since year 2000 without any serious adverse effects. A group of older children and adults in 10 villages will also receive pneumococcal conjugate vaccine. A second group of older children and adults in 11 villages will receive a single dose of meningococcal polysaccharide vaccine.

We will place a thin cotton-tipped soft plastic swab into one of the nostrils to collect some secretions from the throat. This procedure is not painful and is not associated with any risk of injury. It will only cause mild and temporary discomfort to the person. For most of the villagers it will be done only once. For a number of persons it will be done a number of times and we will explain to the parents or participants before it is done.

You and some members of your household will be asked to contribute a small volume of blood, about one spoonful (5 ml) and saliva when the swab is taken. These samples will be tested for the presence of substances that can neutralize the activities of the germs.

You and your family can withdraw from this study at any time. Participation in the study is entirely voluntary and will not interfere with standard healthcare that you and your family would normally receive or with their routine vaccination.

Do you have any questions about the study?

Do you agree to join the study? If you agree the consent form will be read to you before you sign the form.

The field assistant will countersign the consent form to indicate the compound heads understood the explanation and freely gave their consent

Contact information: If you require further information about the study, please contact the following people at the addresses shown below:

1. Dr Richard Adegbola
MRC Laboratories, Fajara
P O Box 273 Banjul. The Gambia
Phone: 495442 (work)

2. Dr Uzochukwu Egere
MRC Laboratories
Phone: 7820343.

Phone: 4496580 (home)
Fax: 4495919

MEDICAL RESEARCH COUNCIL (MRC)/GAMBIAN COMMUNITY PNEUMOCOCCAL CONJUGATE VACCINE TRIAL

CONSENT FORM 1

Consent to join the trial- compound heads

I understand that my family and I have been asked to take part in a trial of two vaccines that prevent meningitis and pneumonia. I understand that if I agree to take part in the trial my family and I will receive one or other of these vaccines with 3 injections for young babies, 2 for older children and 1 for adults. It has been explained to me that these vaccines have been given to many thousands of people and are safe but that they may cause a little pain where they are injected and a little fever. I understand that if I agree for my family to be vaccinated then swabs may be collected from our throats using a small stick with wool on the end one to three times a year (three times in young babies). Some members of the household will be asked to contribute a small volume of blood, about one spoonful (5 ml) and saliva before and after vaccination and subsequently at 3, 12 and 24 months after the completion of the first round of vaccination. I understand that each person will be asked for his or her permission before a throat swab, saliva or blood sample is collected. I understand that participation in the trial is entirely voluntary and will not interfere with standard healthcare that my family would normally receive or with their routine vaccination. The study doctor and nurse field workers will provide free healthcare services to all study participants during the study period. I understand that I or any member of my family can leave the trial at any point without this interfering with their access to healthcare service if they become sick in any way.

I have had an opportunity to ask the MRC field worker who explained the trial to me any questions that I had about the trial.

I agree that my family and/or* I can join the study.

Name...

Signature or thumbprint

I, ..., confirm that I have explained the nature of the

trial to... as set out in the study protocol (SOP no.), that s/he understood what I said, had an opportunity to ask questions and freely gave his/her consent for him/her and/or* his/her family to join the trial.

NAME OF FIELD WORKER...

SIGNATURE..

DATE |__|__|/|__|__|/|__|__|__|__|

and/or* = delete as appropriate

Contact information: If you require further information about the study, please contact the following people at the addresses shown below:

2. Dr Richard Adegbola
   MRC Laboratories, Fajara
   P O Box 273 Banjul. The Gambia
   Phone: 4495442 (work)

2. Dr Uzochukwu Egere
   MRC Laboratories
   Phone:7820343.

MEDICAL RESEARCH COUNCIL (MRC)/GAMBIAN COMMUNITY PNEUMOCOCCAL CONJUGATE VACCINE TRIAL

CONSENT FORM 2

Consent form for subjects from who repeated nasopharyngeal samples will be collected

I have previously given my consent to be vaccinated with one or other of two vaccines that prevent meningitis and pneumonia. I understand that I am now being asked to provide a number of samples from my throat to see the effects of the vaccine on the germs that cause these illnesses and that live in the throat during the next few years. I understand that initially I will be asked to give a sample every month for 6 months and then 3 times a year for 2 years. I understand that collecting these samples is a little uncomfortable but that it will not do any harm. It has been explained to me that I may withdraw from the study at any time.

I have had an opportunity to ask any questions about what participation in the study will involve.

I agree to join the study.

Name ....

Signature or thumbprint

____

I, ..., confirm that I have explained the nature of the

trial to.. as set out in the study protocol (SOP no.), that s/he understood what I said, had an opportunity to ask questions and freely gave his/her consent for to join the trial.

NAME OF FIELD WORKER..

SIGNATURE..

DATE |__|__|/|__|__|/|__|__|__|__|

Contact information: If you require further information about the study, please contact the following people at the addresses shown below:

3. Dr Richard Adegbola
MRC Laboratories, Fajara
P O Box 273 Banjul. The Gambia
Phone: 4495442 (work)
Phone: 4496580 (home)
Fax: 4495919

2. Dr Uzochukwu Egere
MRC Laboratories
Phone: 7820343

MEDICAL RESEARCH COUNCIL (MRC)/GAMBIAN COMMUNITY PNEUMOCOCCAL CONJUGATE VACCINE TRIAL

CONSENT FORM 3

Consent to join the trial- children

I understand I have been asked to permit my child to take part in a trial of two vaccines that prevent meningitis and pneumonia. I understand that if I agree to permit my child to take part in the trial my child will receive one or other of these vaccines with 3 injections for young babies and 2 for older children. It has been explained to me that these vaccines have been given to many thousands of people and are safe but that they may cause a little pain where they are injected and a little fever. I understand that if I agree for my child to be vaccinated then swabs may be collected from his/her throats using a small stick with wool on the end one to three times a year (three times in young babies). I understand that my child may be asked to contribute a small volume of blood, about one spoonful (5 ml) and saliva before and after vaccination and subsequently at 3, 12 and 24 months after the completion of the first round of vaccination. I understand that participation in the trial is entirely voluntary and will not interfere with standard healthcare that my family would normally receive or with their routine vaccination. The study doctor and nurses will provide free healthcare services to all study participants during the study period. I understand that I or any member of my family can leave the trial at any point without this interfering with their access to healthcare service if they become sick in any way.

I have had an opportunity to ask the MRC field worker who explained the trial to me and answers to any questions that I had about the trial.

I agree that my child can join the study.

Name…………………………………………………………………………………………………

Signature or thumbprint……………………………………………………………………………..

Subject ID of child…………………………………………………………………………………...

I, ……………………………………………………., confirm that I have explained the nature of the

trial to……………………………………………………………. as set out in the study protocol (SOP no.), that s/he understood what I said, had an opportunity to ask questions and freely gave his/her consent for him/her and/or* his/her family to join the trial.

NAME OF FIELD
WORKER……………………………………………………………………………….

SIGNATURE…………………………………………………………………………………………
…..

DATE |__|__|/|__|__|/|__|__|__|__|

and/or* = delete as appropriate

Contact information: If you require further information about the study, please contact the following people at the addresses shown below:

Dr Richard Adegbola
MRC Laboratories, Fajara
P O Box 273 Banjul. The Gambia
Phone: 495442 (work)
Phone: 496580 (home)
Fax: 495919

Dr Uzochukwu Egere
MRC Laboratories
Phone: 7820343

MEDICAL RESEARCH COUNCIL (MRC)/GAMBIAN COMMUNITY PNEUMOCOCCAL CONJUGATE VACCINE TRIAL

CONSENT FORM 4

Consent to join the trial- Adults

I understand that I have been asked to take part in a trial of two vaccines that prevent meningitis and pneumonia. I understand that if I agree to take part in the trial I will receive a dose of one or other of these vaccines. It has been explained to me that these vaccines have been given to many thousands of people and are safe but that they may cause a little pain where they are injected and a little fever. I understand that if I agree to be vaccinated then swabs may be collected from our throats using a small stick with wool on the end one to three times a year. I understand that I may be asked to contribute a small volume of blood, about one spoonful (5 ml) and saliva before and after vaccination and subsequently at 3, 12 and 24 months after the completion of the first round of vaccination. I understand that participation in the trial is entirely voluntary and will not interfere with standard healthcare that I would normally receive. The study doctor and nurses will provide free healthcare services to all study participants during the study period. I understand that I can leave the trial at any point without this interfering with my access to healthcare service if I become sick in any way.

I have had an opportunity to ask the MRC field worker who explained the trial to me and answers to questions that I had about the trial.

I agree to join the study.

Name……………………………………………………………………………………………………

Subject ID………………………………………………………………………………………………

Signature or thumbprint

I, ………………………………………………………., confirm that I have explained the nature of the

trial to…………………………………………………………… as set out in the study protocol (SOP no.), that s/he understood what I said, had an opportunity to ask questions and freely gave his/her consent for him/her and/or* his/her family to join the trial.

NAME OF FIELD
WORKER……………………………………………………………………………….

SIGNATURE………………………………………………………………………………………………
…..

DATE |__|__|/|__|__|/|__|__|__|__|

and/or* = delete as appropriate

Contact information: If you require further information about the study, please contact the following people at the addresses shown below:

Dr Richard Adegbola
MRC Laboratories, Fajara
P O Box 273 Banjul. The Gambia
Phone: 495442 (work)
Phone: 496580 (home)
Fax: 495919

Dr Uzochukwu Egere
MRC Laboratories
Phone: 7820343
